# Identifying sexual health concerns, risk-taking and violence using the newly developed SEXIT Adult tool in a Swedish public outpatient sample

**DOI:** 10.1186/s12889-026-28764-5

**Published:** 2026-07-30

**Authors:** Alexis Gainza Solenzal, Kristofer Bjerså, Eva Elmerstig, Sandra Weineland, Johanna Ejblom, Sofia Hammarström

**Affiliations:** 1 Department of Public Health and Community Medicine. Family Medicine, Institute of Medicine, Sahlgrenska Academy, University of Gothenburg, Gothenburg, 405 30 Sweden; 2 Center for Sexual Medicine, Region Västra Götaland, Gothenburg, Sweden; 3 Department of Surgery, Clinical Sciences, Sahlgrenska Academy, University of Gothenburg, Gothenburg, Sweden; 4 Primary Care, Region Västra Götaland, Närhälsan Majorna, Gothenburg, Sweden; 5 Department of Social Work, Centre for Sexology and Sexuality Studies, Malmö University, Malmö, Sweden; 6 Department of Psychology, University of Gothenburg, Gothenburg, Sweden; 7 Research and Development, Primary Health Care, Region Västra Götaland, Borås, Sweden; 8 Knowledge Center for Sexual Health, Region Västra Götaland, Gothenburg, Sweden

**Keywords:** Outpatient care, Sexual health, Sexual behavior, Psychosocial intervention

## Abstract

**Background:**

Sexual health concerns, risk-taking behaviors, and experiences of violence are common among outpatients but are rarely addressed during healthcare consultations. Patients seldom raise these issues themselves, and healthcare professionals (HCPs) often lack the knowledge and confidence to initiate discussions. The SEXual health Identification Tool (SEXIT) was developed to facilitate identification of sexual health concerns, risk-taking behaviors, and experiences of violence. This is the first study to evaluate the use of the SEXIT Adult questionnaire (18+) in a diverse Swedish outpatient population. The study examined the questionnaire’s ability to identify individuals’ needs of further discussion and explored whether responses indicating need for follow-up differed by age, gender identity, sexual orientation, or clinics sexual health specialization.

**Methods:**

Consecutive data collection was conducted using the paper-based SEXIT Adult questionnaire at 10 publicly funded outpatient clinics in Sweden during a three-month period in 2023–2024. The sample comprised 438 patients. Data were collected by 61 HCPs, including physicians, midwives, nurses, psychologists, and social workers. Analyses included descriptive statistics and parametric tests. Differences in the mean number of responses indicating need for follow-up were examined using one-way ANOVA (age, gender identity) and independent t-tests (sexual orientation, clinic specialization).

**Results:**

More than 90% of participants provided at least two responses indicating follow-up. Nearly half (49.1%) reported ongoing sexual problems, and 58.0% reported experiences of sexual violence. Furthermore, 4.4% reported having crossed another person’s sexual boundaries. Patients attending clinics specializing in sexual health reported significantly more responses indicating need for follow-up than those attending non-specialized clinics. Individuals aged 30–54 years reported more such responses than both younger and older age groups. Sexually diverse participants reported more concerns than heterosexual participants.

**Conclusions:**

SEXIT Adult identified a substantial and likely unmet need for discussions about sexual health, risk-taking behaviors, and experiences of violence in outpatient care. The findings highlight the importance of routinely addressing these issues in clinical encounters and underscore the need for knowledgeable, inclusive, and tailored care, particularly for gender and sexually diverse populations.

**Supplementary Information:**

The online version contains supplementary material available at 10.1186/s12889-026-28764-5.

## Background

Shame and embarrassment often hinder patients from disclosing sexual health concerns and experiences of violence to their health care professionals (HCPs) [1–3]. In a large Swedish population survey only 18% of those who reported having sexual health concerns had sought care for those concerns [4]. Only 24% of women and 15% of men in the same cohort reported ever having been asked about their sexual health by an HCP [5]. Similarly, regarding experiences of violence, another Swedish population survey found that only 13% of respondents who had experienced violence had ever been asked about violence by an HCP [6]. Meanwhile, multiple studies have found that sexual health impacts overall health and vice versa; people who experience general well-being generally report positive sexual health, while those who experience somatic or mental health issues report sexual health concerns more often than healthy individuals [7–9]. Furthermore, risk-taking behaviors and experiences of violence have been shown to affect sexual health negatively [10–12].

Addressing sexual health, risk-taking behaviors and experiences of violence (hereafter referred to as SRV) in outpatient consultations may help HCPs identify patients in need of sexual health-informed treatment. While several studies have underscored that it is the responsibility of the HCP to raise questions concerning SRV [2, 13, 14], no studies, to our knowledge, have proposed or evaluated a structured approach for systematically addressing SRV with all patients in an outpatient setting.

### SEXIT – a method for addressing sexual health, risk-taking and violence

**Fig. 1:**
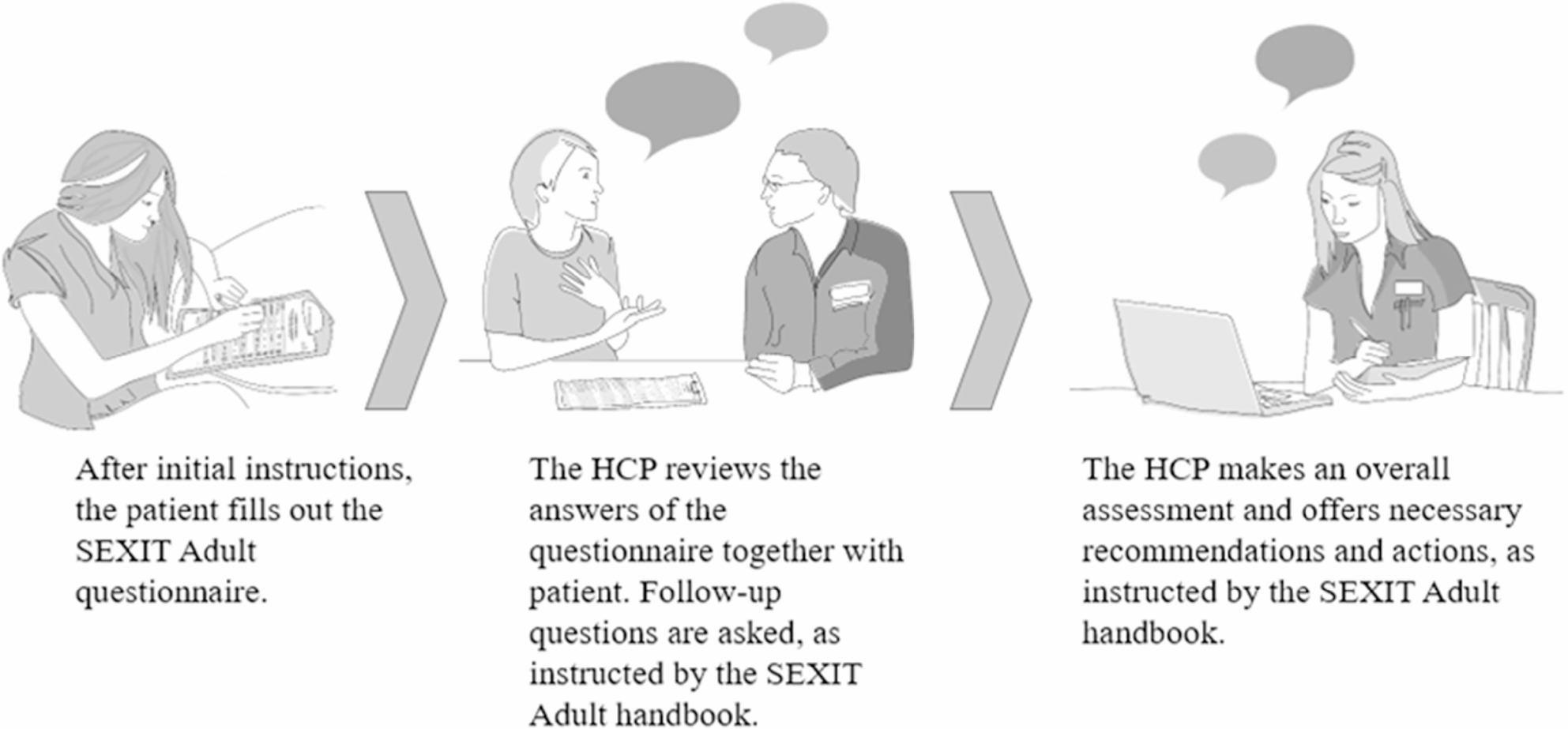
Illustration of the SEXIT Adult method in use during a patient visit

SEXIT (SEXual health Identification Tool) is a method developed to help HCPs address SRV in outpatient consultations. SEXIT comprises (1) an educational program designed for HCPs (2), a patient questionnaire, and (3) a handbook to guide the HCPs in interpreting the patient’s answers to the questionnaire, thus facilitating the conversation on SRV [15]. SEXIT was originally developed to target patients at Swedish youth clinics, aged 13–24. In 2023, the SEXIT Adult method was developed, with 26 questions included in the patient questionnaire. The SEXIT Adult method was adapted through a Delphi-study; 19 academic and/or clinical professionals from diverse areas of expertise took part in the process of selecting and revising the questions to be included in the SEXIT Adult questionnaire (Appendix A). The process consisted of two anonymous rating rounds as well as an online dialog meeting [16]. The questions in the questionnaire function as “gateway questions”, meaning they aim to facilitate further conversation on SRV. Each question has fixed responses (e.g., “yes”, “no”, “I don’t know”, “never”, “sometimes”, etc.), and the SEXIT Adult handbook (Appendix B) instructs the HCP on which responses require follow-up questioning. The process of the SEXIT Adult method during a patient visit is illustrated in Fig. 1.

The youth version of SEXIT has been implemented at many youth clinics in Sweden [17], and the method has been shown to be effective and helpful, both from the perspectives of patients and of the HCPs [18, 19], but the use of the adult version of SEXIT has not yet been explored.

### Domains covered by SEXIT Adult

The SEXIT Adult questionnaire investigates both how patients experience their sexual health and the occurrence of ongoing sexual problems. Many people experience some form of sexual dysfunction during their lifetime. Multiple international studies have estimated that about 30–50% of the general population have experienced sexual problems [20–23]. Furthermore, a correlation has been shown between increased age and sexual dysfunction [24–26]. Research has also shown that sexual dysfunctions are more often experienced by gender and sexually diverse (GSD) individuals than by heterosexual cisgender persons [27, 28].

Several of the questions in the SEXIT Adult questionnaire cover behaviors that may be indicators of sexual risk-taking. Sexual risk-taking, or “high-risk sexual behaviors”, is a complex and questioned concept. There is no consensus on a definition, but most of the previous literature links sexual risk-taking to behaviors that may increase the risk of sexually transmitted infections (STIs) or unintended pregnancies, e.g., condom-less sex and a higher number of casual sexual partners [29]. In this paper, the term “risk-taking behaviors” refers to behaviors that are linked to a statistically increased risk for STIs and unintended pregnancies, as well as behaviors that may lead to negative psychological consequences for oneself and others. Such psychological consequences include experiences of loss of control, self-harm or risk of crossing others’ sexual boundaries. However, it is of the utmost importance to take the full context of the behaviors and experiences of the individual into account.

Research has identified many factors that impact the risk of unintended pregnancies and STIs, such as condom use [30, 31], prior occurrence of an STI [32], use of contraceptives [33], prior unintended pregnancies [34], substance use [35, 36], transactional sex [37–39] and sexual compulsivity/impulsivity [40, 41]. All these factors are covered by questions in the SEXIT Adult questionnaire. Moreover, certain groups have been identified as being at greater risk for unintended pregnancies or STIs, e.g., youths and people belonging to GSD populations [42–44].

One of the questions in the SEXIT Adult questionnaire examines possible paraphilic interests. Prevalence rates of acting out paraphilic interests have varied in different studies and populations and have seemed to depend on the type of paraphilic interest, its risk, and possible harm to oneself and others. For instance, prevalence rates for acting out pedophilic behaviors have ranged from 0.4 to around 4% in different international studies [45–47]. Studies that have examined paraphilic interests and behaviors in both men and women have found that such interests and behaviors are more often experienced by men than by women [46, 48]. However, sexual perpetrators and persons at risk of sexual perpetration have seldom voluntarily sought out treatment [49, 50].

Several of the questions in SEXIT address non-consensual acts, including experiences of violence. Many studies have shown correlations between experiences of subjection to psychological, physical, or sexual violence and a wide range of negative health outcomes [17, 51, 52]. Approximately one third of all women have been subjected to sexual and/or physical violence during their lifetime [53]. The prevalence of male victimization is uncertain [54, 55], but studies have found estimates ranging from 0.3 to 55% regarding sexual abuse, suggesting male victimization has been under-researched [55, 56]. Multiple studies show that individuals belonging to GSD populations are subjected to more violence than heterosexual cisgender persons [57–59].

The SEXIT Adult questionnaire also includes a question on sexual consent communication. Sexual consent refers to the internal will to engage in a sexual act as well as the external communication of this will [60]. Since the # MeToo movement, sexual consent has received increased attention and studies have found that consensual sex is linked to increased sexual satisfaction [61]. Furthermore, a lack of knowledge of sexual consent has been correlated to a risk of both perpetrating and experiencing sexual abuse [62, 63].

### Knowledge gap and aim of this study

Although SRV concerns are common in the general population, these matters are often under-addressed in health care settings [13, 64, 65]. Both patients and HCPs express a willingness to engage in conversations about these topics [2, 13, 66], yet such discussions are frequently hindered by feelings of unease and a lack of appropriate knowledge or tools [13, 64, 65]. Today there is a lack of research on structured approaches to identifying and addressing these concerns within routine health care. Notably, no published studies, as far as we know, have explored these issues among Swedish outpatients specifically. To strengthen the understanding of SEXIT’s practical value, it is important to explore how frequently SEXIT Adult identifies patients who may benefit from follow-up questioning, i.e. in-depth conversations, on SRV. Hence, this study aimed to identify the need for in-depth conversations on SRV among patients from diverse Swedish public outpatient clinics using the newly developed SEXIT Adult method. Furthermore, differences concerning age, gender identity, sexual orientation and clinic category were examined.

## Method

This study was performed by employing a consecutive data collection using paper-based SEXIT Adult questionnaires.

### Sample

Patients scheduled for in-person visits with SEXIT Adult-educated HCPs at ten public outpatient clinics in five different Swedish cities were consecutively recruited for participation in this study, between November 2023 and February 2024. The inclusion criteria for participation were an age of 18 years or older, the ability to read and understand the Swedish language, and written consent to participate in the study. The exclusion criterion was current cognitive impairment resulting in the inability to understand the information about the study.

This research was performed as an explorative study of the SEXIT Adult method, and not as an epidemiological study on SRV. Hence, no power calculation on sample size was performed. Instead, the prospective number of respondents to be recruited was set at 500 in total to have enough responses to be able to analytically explore differences concerning age, gender identity and sexual orientation.

### Data collection

The data collection was performed at the ten public outpatient clinics, of which four specialized in sexual health. The other six clinics consisted of primary care centers, psychiatric clinics, and dependency disorder clinics. The clinics were purposely chosen to obtain a variety of clinics that regularly met adult patients.

**Fig. 2:**
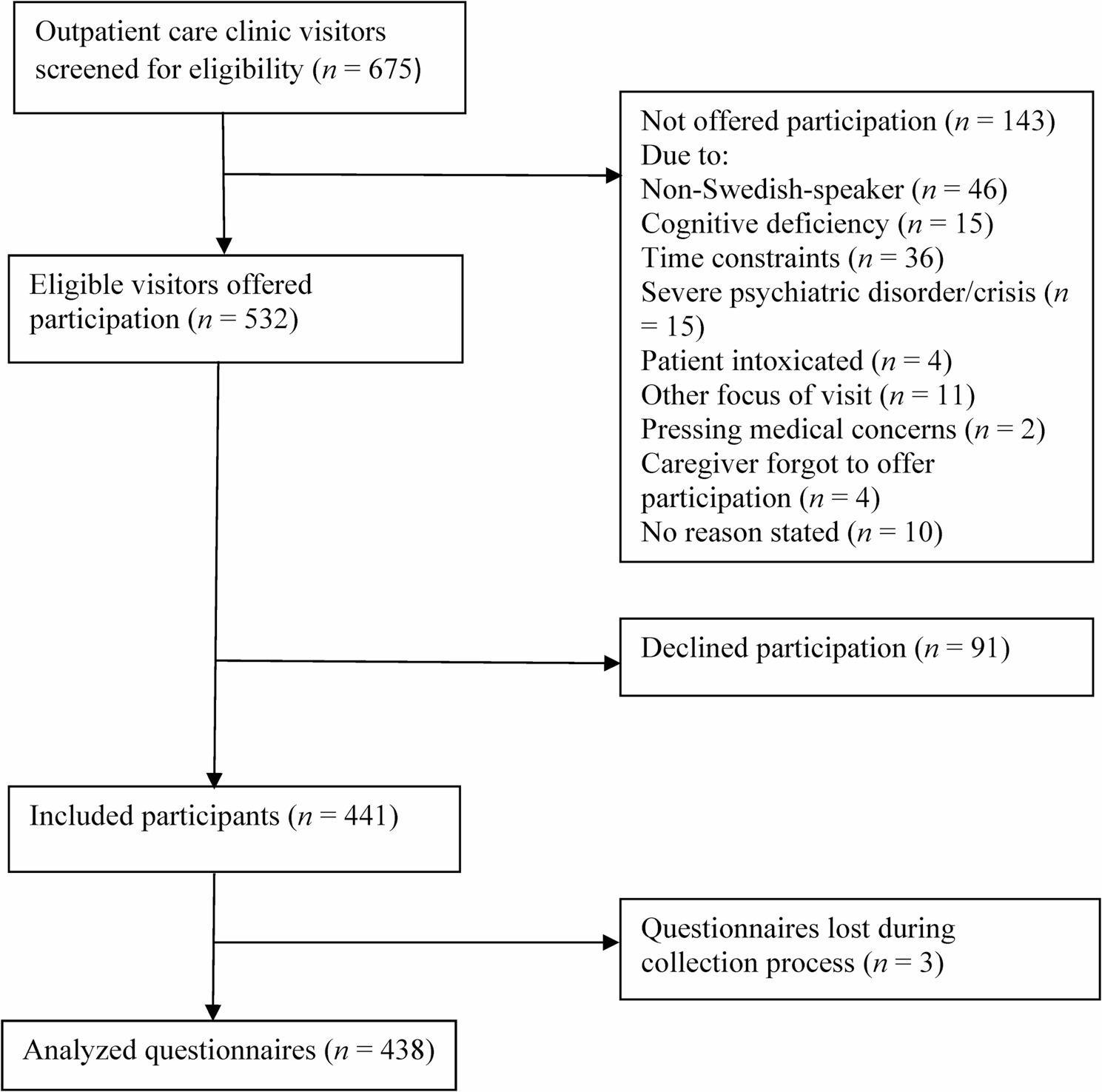
CONSORT flow diagram

In total, 61 HCPs, including physicians, midwives, nurses, psychologists, and social workers, collected the data at the 10 participating clinics. Posters about the study were presented in the waiting room area. Verbal and written consent information was given by the HCPs. The questionnaires were answered by pen and paper by the patients during the visit. Questionnaires were collected by the HCPs during the same visit. Participation was voluntary throughout the outpatient visit; however, withdrawal from the study after the visit was not possible as the questionnaires lacked personal identifiers. The CONSORT flow diagram is presented in Fig. 2. In total, 532 patients were approached to fill out the questionnaire, of whom 441 returned it, resulting in a response rate of 82.9%. However, three questionnaires were lost due to logistical reasons at the clinics.

### Measures

This study used the newly developed [16] SEXIT Adult questionnaire (Appendix A) for the data collection. The questionnaire consists of 26 questions with fixed response options on nominal and ordinal scale levels. The different response options are predetermined as either not indicating risk or health concerns, as requiring further inquiry to assess possible risk or health concerns, or as clearly indicating risk or health concerns. The first three questions in the questionnaire provide demographic information on the patient’s age, gender identity and sexual orientation. The following 14 questions inquire about sexual health satisfaction, sexual problems, consent communication, STI, fertility regulation, transactional sex and potentially harmful or distressing sexual interests and behaviors. Lastly, the questionnaire poses two questions on substance use and seven questions on previous experiences of violence. All 26 questions are designed as “gateway questions”, meaning they aim to cover a broad scope concerning SRV, and help facilitate conversations on the topics where a need for further inquiry is indicated.

### The SEXIT Adult method

In clinical practice, SEXIT Adult is a tool consisting of an HCP training course, the SEXIT Adult questionnaire and the SEXIT Adult handbook. All HCPs participating in the study at the ten clinics undertook the SEXIT Adult training course (see Appendix C for the table of contents of the training course). The training course consists of a one-day program on SRV. The course covers the construction of the SEXIT Adult questionnaire, assessment skills for further management of each question, dealing with difficult situations such as psychologically distressing reactions and judicial concerns, and general skills in communicating sexual health concerns. The course also includes workshops on how to raise questions and follow up patients’ responses while maintaining a norm-critical and sex-positive perspective. This ensured a consistent and informed approach to addressing sensitive topics during the study and aimed to decrease the risk of stigmatizing patients for their sexuality and behaviors. All participating HCPs also had the opportunity to contact a specialist psychologist within the research group to consult on difficult patient matters.

In the SEXIT Adult method as well as the training course, the clinical SEXIT Adult handbook (Appendix B) is a central tool. The handbook includes general information on sexual health-informed health care, a summary of the training course content and assessment management, and in-depth perspectives on all the questions in the SEXIT Adult questionnaire. Responses indicating potential risk or health concerns are highlighted in the handbook with a tick (no indication of risk or health concern), a question mark (possible indication of risk or health concern, indicates follow-up questioning), and an exclamation mark (clear indication for follow-up questioning). In this study, all HCPs received a personal copy of the SEXIT Adult handbook.

### Data analysis

Data was entered in Microsoft Excel and analyzed in Statistical Package for the Social Sciences (SPSS) version 29.0 for Windows.

Age (18–84 years) was analyzed as a non-ordered categorical variable. For this purpose, age was collapsed into three age categories: 18–29 years, 30–54 years, and 55–84 years. Gender identity was compiled into three different categories: female, male and transgender/questioning (including the responses of ‘binary transgender’, ‘non-binary gender’, ‘do not wish to categorize myself by gender’, ‘other’ and ‘don’t know’). Furthermore, sexual orientation was compiled into two categories: heterosexual and LGBQA+ (including responses of ‘homosexual’, ‘bisexual’, ‘queer’, ‘asexual’, ‘pansexual’, ‘do not wish to categorize myself by sexual orientation’, ‘don’t know’ or ‘other’).

Descriptive statistics were reported by the mean, range (min-max), standard deviation (SD), and frequencies as percentages. Differences in the distribution frequencies of nominal variables were analyzed using Pearson’s chi-square test to assess associations between sexual orientation and type of clinic, and between sexual orientation, age group, and gender identity. Differences of sexual orientation within clinics specializing in sexual health were analyzed with Fisher’s exact test.

SEXIT Adult responses indicating need for follow-up questioning, thus suggesting risk or health concerns, were explored. A variable of the total number of questions that received a response indicating follow-up questioning was created, i.e., answers that showed a need for further dialogue on SRV, as instructed by the SEXIT Adult handbook. As age, gender identity and sexual orientation were considered independent variables, the responses indicating follow-up questioning-variable ranged from 0 to 23, i.e., based on the remaining 23 questions in the SEXIT Adult questionnaire.

A one-way ANOVA was used to analyze differences in mean responses indicating follow-up questioning in relation to age and gender identity. Post hoc testing was performed using Tukey’s HSD Test for multiple comparisons to give multiplicity-adjusted mean differences with 95% CI for all levels of age and gender identity. An independent Student t-test was used to analyze differences in mean responses indicating follow-up questioning in relation to sexual orientation and clinic category. Effect sizes were calculated as Eta-squared for age and gender identity and as Cohen’s d for sexual orientation and clinic category. The level of statistical significance was set at p(α) < 0.05.

## Results

### Demographics

The analyzed study population consisted of 438 participants: 217 from the four clinics specializing in sexual health and 221 from the six clinics not specializing in sexual health. Ages of the participants varied between 18 and 84 with a total mean age of 33.9 years. The 18–29 age group was largest, 51.5%, followed by 30–54, 35.1%, and 55–84, 13.4%. Most participants, 59.6%, identified as female, while 33.6% identified as male and 6.8% as transgender/questioning. Heterosexual orientation was most common among the participants, 64.8%, and 35.2% stated an orientation within the LGBQA+ orientations. Demographics stratified by clinic category are presented in Table 1.

**Table 1:** Demographics stratified by clinic category

		Clinic category		*p*.
		Specializing in sexual health	Not specializing in sexual health	
Age	Mean (SD; Min-Max)	28.3 (9.2;18–71)	39.6 (17.1;18–84)	< 0.001
	18–29	66.0% (*n* = 138)	37.1% (*n* = 78)	< 0.001
	30–54	30.6% (*n* = 64)	39.5% (*n* = 83)	
	55–84	3.3% (*n* = 7)	23.3% (*n* = 49)	
Gender identity	Female	54.4% (*n* = 118)	64.7% (*n* = 143)	0.084
	Male	38.2% (*n* = 83)	29.0% (*n* = 64)	
	Transgender and Questioning*	7.4% (*n* = 16)	6.3% (*n* = 14)	
Sexual orientation	Heterosexual	60.2% (*n* = 130)	69.2% (*n* = 153)	0.048
	LGBQA+**	39.8% (*n* = 86)	30.8% (*n* = 68)	

*Transgender/Questioning includes binary transgender, non-binary gender, those not willing to categorize themselves by gender, and those who selected “other” or “don’t know”

**LGBQA+ includes homosexual, bisexual, queer, asexual, pansexual, those not willing to categorize themselves by sexual orientation, and those who selected “other” or “don’t know”

### Descriptive statistics

Apart from age, gender identity and sexual orientation as presented above, the SEXIT Adult questionnaire encompasses a total of 23 questions designed to indicate SRV concerns. A summary of the collected questionnaires in this study revealed that more than 90% of the participants gave two or more responses requiring follow-up among the 23 SEXIT Adult questions, and nearly 59% gave five or more such responses.

Descriptive statistics for all the SEXIT Adult questions are presented in Table 2. More than half of the participants, 55.8%, expressed that they were satisfied with their sexual health, and 33.6% that they were dissatisfied. Nearly half of the respondents, 49.1%, reported having an ongoing sexual problem, and 25.7% of respondents reported having experienced an STI during their lifetime. Loss of control of sexual impulses was reported by 17.2%. Concerning experiences of transactional sex, 10.8% reported having received some form of reimbursement for sexual acts, 2.5% responded that they had given reimbursement, and almost 11.9% had experienced sexual arousal toward something that they perceived as harmful to themselves or others. Almost one fourth, 24.6%, of respondents reported experiencing difficulties concerning sexual consent communication, 58% of participants indicated experiences of some form of sexual violence, and 4.1% indicated that they had subjected others to sexual violence.

**Table 2:** All SEXIT Adult questionnaire responses stratified by age, gender identity and sexual orientation

		Age			Gender identity			Sexual orientation	
		18–29	30–54	55–84	Female	Male	Transgender /Questioning	LGBQA+	Heterosexual
Are you satisfied with your sexual health? (*n* = 437)	Yes	60.6% (*n* = 131)	49.7% (*n* = 73)	60.0% (*n* = 33)	54.6% (*n* = 142)	59.9% (*n* = 88)	46.7% (*n* = 14)	46.1% (*n* = 71)	61.3% (*n* = 173)
	No*	28.2% (*n* = 61)	40.8% (*n* = 60)	30.9% (*n* = 17)	35.0% (*n* = 91)	30.6% (*n* = 45)	36.7% (*n* = 11)	43.5% (*n* = 67)	28.0% (*n* = 79)
	I don’t know*	11.1% (*n* = 24)	9.5% (*n* = 14)	9.1% (*n* = 5)	10.4% (*n* = 27)	9.5% (*n* = 14)	16.7% (*n* = 5)	10.4% (*n* = 16)	10.6% (*n* = 30)
	Responses indicating follow-up questioning	39.3% (*n* = 85)	50.3% (*n* = 74)	40% (*n* = 22)	45.4% (*n* = 118)	40.1% (*n* = 59)	53.4% (*n* = 16)	53.9% (*n* = 83)	38.6% (*n* = 109)
Do you have any sexual problems? (*n* = 434)	Yes*	44.6% (*n* = 95)	52.7% (*n* = 77)	53.6% (*n* = 30)	49.0% (*n* = 128)	51.0% (*n* = 74)	39.3% (*n* = 11)	60.3% (*n* = 91)	42.9% (*n* = 121)
	No	49.3% (*n* = 105)	44.5% (*n* = 65)	44.6% (*n* = 25)	47.1% (*n* = 123)	44.8% (*n* = 65)	53.6% (*n* = 15)	35.1% (*n* = 53)	53.2% (*n* = 150)
	I don’t know*	6.1% (*n* = 13)	2.7% (*n* = 4)	4.3% (*n* = 1)	3.8% (*n* = 10)	4.1% (*n* = 6)	7.1% (*n* = 2)	4.6% (*n* = 7)	3.9% (*n* = 11)
	Responses indicating follow-up questioning	50.7% (108)	55.4% (*n* = 81)	57.9% (*n* = 31)	52.8% (*n* = 138)	55.1% (*n* = 80)	46.4% (*n* = 13)	64.9% (*n* = 98)	46.8% (*n* = 132)
How often do you and your partners use condoms as protection against sexually transmitted diseases? (*n* = 434)	Always	14.6% (*n* = 31)	7.5% (*n* = 11)	5.5% (*n* = 3)	10.5% (*n* = 27)	12.9% (*n* = 19)	6.9% (*n* = 2)	14.4% (*n* = 22)	9.3% (*n* = 26)
	Sometimes*	28.6% (*n* = 61)	25.9% (*n* = 38)	12.7% (*n* = 7)	22.5% (*n* = 58)	31.3% (*n* = 46)	17.2% (*n* = 5)	32.0% (*n* = 49)	21.4% (*n* = 60)
	Never*	35.2% (*n* = 75)	38.8% (*n* = 57)	32.7% (*n* = 18)	36.8% (*n* = 95)	38.1% (*n* = 56)	13.8% (*n* = 4)	23.5% (*n* = 36)	42.5% (*n* = 119)
	I don’t know*	0.5% (*n* = 1)	0.0% (*n* = 0)	0.0% (*n* = 0)	0.4% (*n* = 1)	0.0% (*n* = 0)	0.0% (*n* = 0)	0.0% (*n* = 0)	0.4% (*n* = 1)
	Not applicable**	21.1% (*n* = 45)	27.9% (*n* = 41)	49.1% (*n* = 27)	29.8% (*n* = 77)	17.7% (*n* = 26)	62.1% (*n* = 18)	30.1% (*n* = 46)	26.4% (*n* = 74)
	Responses indicating follow-up questioning	64.3% (*n* = 137)	64.7% (*n* = 95)	45.4% (*n* = 25)	59.7% (*n* = 154)	69.4% (*n* = 102)	31% (*n* = 9)	55.5% (*n* = 85)	64.3% (*n* = 180)
Do you have, or have you had, chlamydia, gonorrhea, syphilis, hepatitis, or HIV? (*n* = 436)	Yes*	17.7% (*n* = 38)	33.3% (*n* = 49)	32.7% (*n* = 18)	30.0% (*n* = 78)	19.2% (*n* = 28)	20.0% (*n* = 6)	31.2% (*n* = 48)	22.8% (*n* = 64)
	No	75.8% (*n* = 163)	61.2% (*n* = 90)	58.2% (*n* = 32)	65.0% (*n* = 169)	74.0% (*n* = 108)	66.7% (*n* = 20)	62.3% (*n* = 96)	71.2% (*n* = 200)
	I don’t know*	4.7% (*n* = 10)	4.1% (*n* = 6)	3.6% (*n* = 2)	2.7% (*n* = 7)	6.2% (*n* = 9)	6.7% (*n* = 2)	5.2% (*n* = 8)	3.6% (*n* = 10)
	Not applicable**	1.9% (*n* = 4)	1.4% (*n* = 2)	5.5% (*n* = 3)	2.3% (*n* = 6)	0.7% (*n* = 1)	6.7% (*n* = 2)	1.3% (*n* = 2)	2.5% (*n* = 7)
	Responses indicating follow-up questioning	22.4% (*n* = 48)	37.4% (*n* = 55)	36.3% (*n* = 20)	32.7% (*n* = 85)	25.4% (*n* = 37)	26.7% (*n* = 8)	36.4% (*n* = 56)	26.4% (*n* = 74)
How often do you and your partners use birth control? (*n* = 430)	Always	63.7% (*n* = 137)	39.4% (*n* = 56)	3.6% (*n* = 2)	50.4% (*n* = 131)	42.1% (*n* = 59)	33.3% (*n* = 10)	47.7% (*n* = 71)	45.7% (*n* = 128)
	Sometimes*	12.1% (*n* = 26)	14.8% (*n* = 21)	1.8% (*n* = 1)	11.2% (*n* = 29)	14.3% (*n* = 20)	3.3% (*n* = 1)	7.4% (*n* = 11)	13.9% (*n* = 39)
	Never*	5.1% (*n* = 11)	12% (*n* = 17)	32.7% (*n* = 18)	9.2% (*n* = 24)	17.1% (*n* = 24)	6.7% (*n* = 2)	8.7% (*n* = 13)	13.2% (*n* = 37)
	I don’t know*	1.4% (*n* = 3)	0.7% (*n* = 1)	0.0% (*n* = 0)	0.0% (*n* = 0)	2.1% (*n* = 3)	3.3% (*n* = 1)	0.7% (*n* = 1)	1.1% (*n* = 3)
	Not applicable**	17.7% (*n* = 38)	33.1% (*n* = 47)	61.8% (*n* = 34)	29.2% (*n* = 76)	24.3% (*n* = 34)	53.3% (*n* = 16)	35.6% (*n* = 53)	26.1% (*n* = 73)
	Responses indicating follow-up questioning	18.6%(*n* = 40)	28.2% (*n* = 39)	34.5% (*n* = 19)	20.4% (*n* = 53)	33.5% (*n* = 47)	10.0% (*n* = 3)	16.8% (*n* = 25)	28.2% (*n* = 79)
Have you or a partner had an unplanned pregnancy? (*n* = 433)	Yes*	7.5% (*n* = 16)	28.6% (*n* = 42)	36.3% (*n* = 20)	23.3% (*n* = 60)	12.4% (*n* = 18)	6.7% (*n* = 2)	12.6% (*n* = 19)	21.7% (*n* = 61)
	No	82.2% (*n* = 175)	63.9% (*n* = 94)	50.9% (*n* = 28)	68.6% (*n* = 177)	78.6% (*n* = 114)	63.3% (*n* = 19)	74.8% (*n* = 113)	69.8% (*n* = 196)
	I don’t know*	1.4% (*n* = 3)	1.4% (*n* = 2)	0.0% (*n* = 0)	0.8% (*n* = 2)	3.4% (*n* = 5)	0.0% (*n* = 0)	0.7% (*n* = 1)	2.1% (*n* = 6)
	Not applicable**	8.9% (*n* = 19)	6.1% (*n* = 9)	12.7% (*n* = 7)	7.4% (*n* = 19)	5.5% (*n* = 8)	30% (*n* = 9)	11.9% (*n* = 18)	6.4% (*n* = 18)
	Responses indicating follow-up questioning	8.9% (*n* = 19)	30.0% (*n* = 44)	38.3% (*n* = 20)	24.1% (*n* = 62)	15.8% (*n* = 23)	6.7% (*n* = 2)	13.3% (*n* = 20)	23.8% (*n* = 67)
Have you ever been subjected to non-consensual sexual acts (sex you didn’t want)? (*n* = 436)	Yes*	57.2% (*n* = 123)	64.6% (*n* = 95)	40.0% (*n* = 22)	73.9% (*n* = 193)	29.0% (*n* = 42)	60.0% (*n* = 18)	75.3% (*n* = 116)	48.8% (*n* = 137)
	No	39.5% (*n* = 85)	33.3% (*n* = 49)	60.0% (*n* = 33)	24.9% (*n* = 65)	66.9% (*n* = 97)	33.3% (*n* = 10)	21.4% (*n* = 33)	49.1% (*n* = 138)
	I don’t know*	3.3% (*n* = 7)	2.0% (*n* = 3)	0.0% (*n* = 0)	1.1% (*n* = 3)	4.1% (*n* = 6)	6.7% (*n* = 2)	3.2% (*n* = 5)	2.1% (*n* = 6)
	Responses indicating follow-up questioning	60.5% (*n* = 130)	66.6% (*n* = 98)	40% (*n* = 22)	75% (*n* = 196)	33.1% (*n* = 48)	66.7% (*n* = 20)	78.5% (*n* = 121)	50.9% (*n* = 143)
Have you ever subjected someone else to non-consensual sexual acts (sex they didn’t want)? (*n* = 437)	Yes*	2.3% (*n* = 5)	5.4% (*n* = 8)	5.5% (*n* = 3)	3.1% (*n* = 8)	5.5% (*n* = 8)	6.7% (*n* = 2)	5.8% (*n* = 9)	3.2% (*n* = 9)
	No	91.2% (*n* = 197)	89.9% (*n* = 132)	92.7% (*n* = 51)	93.5% (*n* = 244)	87.0% (*n* = 127)	83.3% (*n* = 25)	86.4% (*n* = 133)	92.9% (*n* = 262)
	I don’t know*	6.5% (*n* = 14)	4.8% (*n* = 7)	1.8% (*n* = 1)	3.4% (*n* = 9)	7.5% (*n* = 11)	10.0% (*n* = 3)	7.8% (*n* = 12)	3.9% (*n* = 11)
	Responses indicating follow-up questioning	8.8% (*n* = 19)	10.2% (*n* = 15)	7.3% (*n* = 4)	6.5% (*n* = 17)	13% (*n* = 19)	16.7% (*n* = 5)	13.6% (*n* = 21)	7.1% (*n* = 20)
Do you find it difficult to communicate about consent in sexual situations? (*n* = 435)	Yes*	26.5% (*n* = 57)	28.8% (*n* = 42)	9.1% (*n* = 5)	29.6% (*n* = 77)	14.5% (*n* = 21)	30.0% (*n* = 9)	40.3% (*n* = 62)	15.7% (*n* = 44)
	No	66.0% (*n* = 142)	63.0% (*n* = 92)	81.8% (*n* = 45)	61.9% (*n* = 161)	80.0% (*n* = 116)	53.3% (*n* = 16)	48.7% (*n* = 75)	77.9% (*n* = 218)
	I don’t know*	7.4% (*n* = 16)	8.2% (*n* = 12)	9.1% (*n* = 5)	8.5% (*n* = 22)	5.5% (*n* = 8)	16.7% (*n* = 5)	11.0% (*n* = 17)	6.4% (*n* = 18)
	Responses indicating follow-up questioning	33.9% (*n* = 73)	37% (*n* = 54)	18.2% (*n* = 10)	38.1% (*n* = 99)	20% (*n* = 29)	46.7% (*n* = 14)	51.3% (*n* = 79)	22.1% (*n* = 62)
Have you used sex to handle difficult feelings, or to intentionally harm yourself? (*n* = 433)	Yes*	29.6% (*n* = 63)	35.6% (*n* = 52)	14.5% (*n* = 8)	30.7% (*n* = 80)	23.9% (*n* = 34)	50.0% (*n* = 15)	45.8% (*n* = 70)	21.1% (*n* = 59)
	No	63.4% (*n* = 135)	57.5% (*n* = 84)	81.8% (*n* = 45)	61.7% (*n* = 161)	71.8% (*n* = 102)	43.3% (*n* = 13)	43.1% (*n* = 66)	74.9% (*n* = 209)
	I don’t know*	7.0% (*n* = 15)	6.8% (*n* = 10)	3.6% (*n* = 2)	7.7% (*n* = 20)	4.2% (*n* = 6)	6.7% (*n* = 2)	11.1% (*n* = 17)	3.9% (*n* = 11)
	Responses indicating follow-up questioning	36.6% (*n* = 78)	42.4% (*n* = 62)	18.1% (*n* = 10)	38.4% (*n* = 100)	28.1% (*n* = 40)	56.7% (*n* = 17)	56.9% (*n* = 87)	25.0% (*n* = 70)
Do you lose control over your sexuality and do things you regret or that worry you? (*n* = 436)	Yes*	16.3% (*n* = 35)	22.4% (*n* = 33)	3.6% (*n* = 2)	12.6% (*n* = 33)	23.4% (*n* = 34)	26.7% (*n* = 8)	26.0% (*n* = 40)	12.5% (*n* = 35)
	No	78.6% (*n* = 169)	72.8% (*n* = 107)	78.4% (*n* = 51)	83.1% (*n* = 217)	71.0% (*n* = 103)	66.7% (*n* = 20)	67.5% (*n* = 104)	83.6% (*n* = 235)
	I don’t know*	5.1% (*n* = 11)	4.8% (*n* = 7)	3.6% (*n* = 2)	4.2% (*n* = 11)	5.5% (*n* = 8)	6.7% (*n* = 2)	6.5% (*n* = 10)	3.9% (*n* = 11)
	Responses indicating follow-up questioning	21.4% (*n* = 46)	27.2% (*n* = 40)	7.2% (*n* = 4)	16.8% (*n* = 44)	28.9% (*n* = 42)	33.4% (*n* = 10)	32.5% (*n* = 50)	16.4% (*n* = 46)
Have you ever received compensation for performing sexual acts? (*n* = 435)	Yes*	8.4% (*n* = 18)	17.8% (*n* = 26)	1.8% (*n* = 1)	12.7% (*n* = 33)	4.8% (*n* = 7)	24.1% (*n* = 7)	20.9% (*n* = 32)	5.3% (*n* = 15)
	No	90.7% (*n* = 195)	80.1% (*n* = 117)	98.2% (*n* = 54)	85.8% (*n* = 223)	94.5% (*n* = 138)	75.9% (*n* = 22)	77.1% (*n* = 118)	94.0% (*n* = 264)
	I don’t know*	0.9% (*n* = 2)	2.1% (*n* = 3)	0.0% (*n* = 0)	1.5% (*n* = 4)	0.7% (*n* = 1)	0.0% (*n* = 0)	2.0% (*n* = 3)	0.7% (*n* = 2)
	Responses indicating follow-up questioning	9.3% (*n* = 20)	19.9% (*n* = 29)	1.8% (*n* = 1)	14.2% (*n* = 37)	5.5% (*n* = 8)	24.1% (*n* = 7)	22.9% (*n* = 35)	6.0% (*n* = 17)
Have you ever given someone compensation for sexual acts in Sweden or abroad? (*n* = 436)	Yes*	1.4% (*n* = 3)	2.0% (*n* = 3)	5.5% (*n* = 3)	1.2% (*n* = 3)	5.5% (*n* = 8)	0.0% (*n* = 0)	4.5% (*n* = 7)	1.4% (*n* = 4)
	No	98.6% (*n* = 212)	97.3% (*n* = 143)	94.5% (*n* = 52)	98.8% (*n* = 257)	93.8% (*n* = 137)	100% (*n* = 30)	95.5% (*n* = 147)	98.2% (*n* = 276)
	I don’t know*	0.0% (*n* = 0)	0.7% (*n* = 1)	0.0% (*n* = 0)	0.0% (*n* = 0)	0.7% (*n* = 1)	0.0% (*n* = 0)	0.0% (*n* = 0)	0.4% (*n* = 1)
	Responses indicating follow-up questioning	1.4% (*n* = 3)	2.7% (*n* = 4)	5.5% (*n* = 3)	1.2% (*n* = 3)	6.2% (*n* = 9)	0.0% (*n* = 0)	4.5% (*n* = 7)	1.8% (*n* = 5)
Have you ever been sexually aroused by something that could be harmful to you or others? (*n* = 436)	Yes*	10.2% (*n* = 22)	17.1% (*n* = 25)	1.8% (*n* = 1)	10.0% (*n* = 26)	11.0% (*n* = 16)	33.3% (*n* = 10)	21.4% (*n* = 33)	6.8% (*n* = 19)
	No	85.6% (*n* = 185)	76.7% (*n* = 112)	98.2% (*n* = 54)	85.4% (*n* = 222)	85.6% (*n* = 125)	60.0% (*n* = 18)	72.1% (*n* = 111)	90.0% (*n* = 253)
	I don’t know*	4.2% (*n* = 9)	6.2% (*n* = 9)	0.0% (*n* = 0)	4.6% (*n* = 12)	3.4% (*n* = 5)	6.7% (*n* = 2)	6.5% (*n* = 10)	3.2% (*n* = 9)
	Responses indicating follow-up questioning	14.4% (*n* = 31)	23.3% (*n* = 34)	1.8% (*n* = 1)	14.6% (*n* = 38)	14.4% (*n* = 21)	40% (*n* = 12)	27.9% (*n* = 43)	10.0% (*n* = 28)
Are you, or is someone close to you, concerned about how much alcohol you drink? (*n* = 432)	Yes*	7.5% (*n* = 16)	13.0% (*n* = 19)	25.0% (*n* = 14)	14.7% (*n* = 38)	7.6% (*n* = 11)	6.9% (*n* = 2)	12.5% (*n* = 19)	11.5% (*n* = 32)
	No	86.9% (*n* = 185)	83.6% (*n* = 122)	69.6%% (*n* = 39)	80.6% (*n* = 208)	86.9% (*n* = 126)	93.1% (*n* = 27)	83.6% (*n* = 127)	83.5% (*n* = 233)
	I don’t know*	5.6% (*n* = 12)	3.4% (*n* = 5)	5.4% (*n* = 3)	4.7% (*n* = 12)	5.5% (*n* = 8)	0.0% (*n* = 0)	3.9% (*n* = 6)	5.0% (*n* = 14)
	Responses indicating follow-up questioning	13.1% (*n* = 28)	16.4% (*n* = 24)	30.4% (*n* = 17)	19.4% (*n* = 50)	13.1% (*n* = 19)	6.9% (*n* = 2)	16.4% (*n* = 25)	16.5% (*n* = 46)
Have you taken drugs during the past year? (*n* = 435)	Yes*	17.6% (*n* = 38)	17.8% (*n* = 26)	5.4% (*n* = 3)	13.5% (*n* = 35)	20.0% (*n* = 29)	23.3% (*n* = 7)	26.3% (*n* = 40)	10.6% (*n* = 30)
	No	82.4% (*n* = 178)	82.2% (*n* = 120)	94.6% (*n* = 53)	86.5% (*n* = 225)	80.0% (*n* = 116)	76.7% (*n* = 23)	73.7% (*n* = 112)	89.4% (*n* = 252)
	Responses indicating follow-up questioning	17.6% (*n* = 38)	17.8% (*n* = 26)	5.4% (*n* = 3)	13.5% (*n* = 35)	20.0% (*n* = 29)	23.3% (*n* = 7)	26.3% (*n* = 40)	10.6% (*n* = 30)
Has anyone ever restricted or controlled you? (*n* = 435)	Yes*	27.6% (*n* = 59)	33.3% (*n* = 49)	14.3% (*n* = 8)	32.8% (*n* = 85)	16.4% (*n* = 24)	40.0% (*n* = 12)	37.9% (*n* = 58)	22.4% (*n* = 63)
	No	67.7% (*n* = 145)	61.2% (*n* = 90)	83.9% (*n* = 47)	64.1% (*n* = 166)	78.1% (*n* = 114)	50.0% (*n* = 15)	54.9% (*n* = 84)	74.7% (*n* = 210)
	I don’t know*	4.7% (*n* = 10)	5.4% (*n* = 8)	1.8% (*n* = 1)	3.1% (*n* = 8)	5.5% (*n* = 8)	10.0% (*n* = 3)	7.2%(*n* = 11)	2.8% (*n* = 8)
	Responses indicating follow-up questioning	32.3% (*n* = 69)	38.8% (*n* = 57)	16.1% (*n* = 9)	35.9% (*n* = 93)	21.9% (*n* = 32)	50.0% (*n* = 15)	45.1% (*n* = 69)	25.3% (*n* = 71)
Have you ever restricted or controlled someone else? (*n* = 435)	Yes*	3.7% (*n* = 8)	2.0% (*n* = 3)	1.8% (*n* = 1)	2.7% (*n* = 7)	2.0% (*n* = 3)	10.0% (*n* = 3)	5.8% (*n* = 9)	1.4% (*n* = 4)
	No	89.8% (*n* = 193)	93.2% (*n* = 137)	98.2% (*n* = 55)	93.5% (*n* = 244)	91.8% (*n* = 134)	83.3% (*n* = 25)	90.3% (*n* = 139)	93.3% (*n* = 263)
	I don’t know*	6.5% (*n* = 14)	4.8% (*n* = 7)	0.0% (*n* = 0)	3.8% (*n* = 10)	6.2% (*n* = 9)	6.7%(*n* = 2)	3.9% (*n* = 6)	5.3% (*n* = 15)
	Responses indicating follow-up questioning	10.2% (*n* = 22)	6.8% (*n* = 10)	1.8% (*n* = 1)	6.5% (*n* = 17)	8.2% (*n* = 12)	16.7% (*n* = 5)	9.7% (*n* = 15)	6.7% (*n* = 19)
Have you ever been subjected to psychological violence or a threat of violence? (*n* = 437)	Yes*	40.5% (*n* = 87)	51.0% (*n* = 74)	30.4% (*n* = 17)	46.3% (*n* = 120)	36.3% (*n* = 53)	53.3% (*n* = 16)	53.9% (*n* = 83)	37.9% (*n* = 106)
	No	52.6% (*n* = 113)	47.6% (*n* = 69)	69.6% (*n* = 39)	49.0% (*n* = 127)	61.0% (*n* = 89)	40.0% (*n* = 12)	39.6% (*n* = 61)	59.3% (*n* = 166)
	I don’t know*	7.0% (*n* = 15)	1.4% (*n* = 2)	0.0% (*n* = 0)	4.6% (*n* = 12)	2.7% (*n* = 4)	6.7% (*n* = 2)	6.5% (*n* = 10)	2.9% (*n* = 8)
	Responses indicating follow-up questioning	47.4% (*n* = 102)	52.4% (*n* = 76)	30.4% (*n* = 17)	51.0% (*n* = 132)	39.0% (*n* = 57)	60.0% (*n* = 18)	60.4% (*n* = 93)	40.7% (*n* = 114)
Have you ever subjected someone to psychological violence or a threat of violence? (*n* = 435)	Yes*	6.5% (*n* = 14)	10.3% (*n* = 15)	1.8% (*n* = 1)	5.4% (*n* = 14)	7.5% (*n* = 11)	20.0% (*n* = 6)	11.8% (*n* = 18)	4.6% (*n* = 13)
	No	87.9% (*n* = 188)	86.3% (*n* = 126)	96.4% (*n* = 54)	90.7% (*n* = 235)	87.7% (*n* = 128)	66.7% (*n* = 20)	81.0% (*n* = 124)	91.8% (*n* = 258)
	I don’t know*	5.6% (*n* = 12)	3.4% (*n* = 5)	1.8% (*n* = 1)	3.9% (*n* = 10)	4.8% (*n* = 7)	13.3% (*n* = 4)	7.2% (*n* = 11)	3.6% (*n* = 10)
	Responses indicating follow-up questioning	12.1% (*n* = 26)	13.7% (*n* = 20)	3.6% (*n* = 2)	9.3% (*n* = 24)	12.3% (*n* = 18)	33.3% (*n* = 10)	19.0% (*n* = 29)	8.2% (*n* = 23)
Have you ever been subjected to physical violence? (*n* = 435)	Yes*	33.0% (*n* = 71)	43.1% (*n* = 63)	36.4% (*n* = 20)	38.5% (*n* = 100)	32.4% (*n* = 47)	46.7% (*n* = 14)	49.7% (*n* = 76)	29.9% (*n* = 84)
	No	64.2% (*n* = 138)	56.2% (*n* = 82)	63.6% (*n* = 35)	60.4% (*n* = 157)	64.8% (*n* = 94)	50.0% (*n* = 15)	46.4% (*n* = 71)	69.4% (*n* = 195)
	I don’t know*	2.8% (*n* = 6)	0.7% (*n* = 1)	0.0% (*n* = 0)	1.1% (*n* = 3)	2.8% (*n* = 4)	3.3% (*n* = 1)	3.9% (*n* = 6)	0.7% (*n* = 2)
	Responses indicating follow-up questioning	35.8% (*n* = 77)	43.8% (*n* = 64)	36.4% (*n* = 20)	39.6% (*n* = 103)	35.2% (*n* = 51)	50.0% (*n* = 15)	53.6% (*n* = 82)	30.6% (*n* = 86)
Have you ever subjected someone to physical violence? (*n* = 435)	Yes*	13.5% (*n* = 29)	17.8% (*n* = 26)	12.5% (*n* = 7)	13.1% (*n* = 34)	18.5% (*n* = 27)	20.0% (*n* = 6)	17.1% (*n* = 26)	14.2% (*n* = 40)
	No	84.6% (*n* = 181)	80.8% (*n* = 118)	87.5% (*n* = 49)	85.7% (*n* = 222)	80.1% (*n* = 117)	73.3% (*n* = 22)	80.3% (*n* = 122)	84.8% (*n* = 239)
	I don’t know*	1.9% (*n* = 4)	1.4% (*n* = 2)	0.0% (*n* = 0)	1.2% (*n* = 3)	1.4% (*n* = 2)	6.7% (*n* = 2)	2.6% (*n* = 4)	1.1% (*n* = 3)
	Responses indicating follow-up questioning	15.4% (*n* = 33)	29.2% (*n* = 28)	12.5% (*n* = 7)	14.3% (*n* = 37)	19.9% (*n* = 29)	26.7% (*n* = 8)	19.7% (*n* = 30)	15.2% (*n* = 43)
When you were growing up, did you ever see anyone in your family being subjected to psychological, physical, or sexual violence? (*n* = 437)	Yes*	28.8% (*n* = 62)	42.6% (*n* = 63)	30.4% (*n* = 17)	36.2% (*n* = 94)	28.6% (*n* = 42)	46.7% (*n* = 14)	40.9% (*n* = 63)	30.9% (*n* = 87)
	No	63.7% (*n* = 137)	51.7% (*n* = 76)	66.1% (*n* = 37)	57.3% (*n* = 149)	66.0% (*n* = 97)	43.3% (*n* = 13)	48.7% (*n* = 75)	64.9% (*n* = 183)
	I don’t know*	7.4% (*n* = 16)	5.4% (*n* = 8)	3.6% (*n* = 2)	6.5% (*n* = 17)	5.4% (*n* = 8)	10.0% (*n* = 3)	10.4% (*n* = 16)	6.6% (*n* = 12)
	Responses indicating follow-up questioning	36.3% (*n* = 78)	48.3% (*n* = 71)	33.9% (*n* = 19)	42.7% (*n* = 111)	34.0% (*n* = 50)	56.7% (*n* = 17)	51.3% (*n* = 79)	35.1% (*n* = 99)

*These responses were categorized as indicating follow-up questioning, as they required further dialogue between an HCP and patient according to the SEXIT Adult handbook

**Responses of “Not applicable” were not categorized as indicating follow-up questioning although follow-up questions are recommended by the SEXIT Adult handbook

### Comparisons of responses indicating follow-up questioning

Differences in responses indicating follow-up, as instructed by the SEXIT Adult handbook, were analyzed with ANOVA for age and gender identity (see Table 3).

**Table 3:** Results of statistical analysis of comparisons of responses indicating follow-up questioning for age, gender identity, sexual orientation and clinic category

		% (n)	Responses indicating follow-up questioning (Mean (SD))	Statistical analysis (Mean difference (95%CI)*; Effect size**)
Age	18–29	51.5% (*n* = 216)	6.1 (4.0)	ANOVA *p* < 0.001 Mean difference 18–29/30–54: **-**1.4 Mean difference 30–54/55–84: 2.4 Mean difference 18–29/55–84: 0.9 Effect size: 0.039
	30–54	35.1% (*n* = 147)	7.5 (4.5)	
	55–84	13.4% (*n* = 56)	5.1 (3.5)	
Gender identity	Female	59.6% (*n* = 261)	6.7 (4.2)	ANOVA *p* = 0.034 Mean difference Female/Male: 0.8 Mean difference Male/Transgender and Questioning: -1.9 Mean difference Female/Transgender and Questioning: -1.1 Effect size: 0.015
	Male	33.6% (*n* = 147)	5.9 (4.0)	
	Transgender and Questioning***	6.8% (*n* = 30)	7.8 (5.3)	
Sexual orientation	Heterosexual	64.8% (*n* = 283)	5.5 (3.8)	Independent samples test *p* < 0.001 Mean difference: 2.7 Effect size: 0.68
	LGBQA+****	35.2% (*n* = 154)	8.3 (4.5)	
Clinic category	Specializing in sexual health	49.5% (*n* = 217)	7.3 (4.7)	Independent samples test *p* < 0.001 Mean difference: -1.7 Effect size: -0.4
	Not specializing in sexual health	50.5% (*n* = 221)	5.6 (3.5)	

*Mean differences found to be statistically significant in post hoc testing (Tukey’s) for age and gender identity are highlighted

**Effect size was calculated as Eta-squared for age and gender identity, and as Cohen’s d for sexual orientation and clinic category

*** Transgender/Questioning includes binary transgender, non-binary gender, those not willing to categorize themselves by gender, and those who selected “other” or “don’t know”

**** LGBQA+ includes homosexual, bisexual, queer, asexual, pansexual, those not willing to categorize themselves by sexual orientation, and those who selected “other” or “don’t know”

**Fig. 3:**
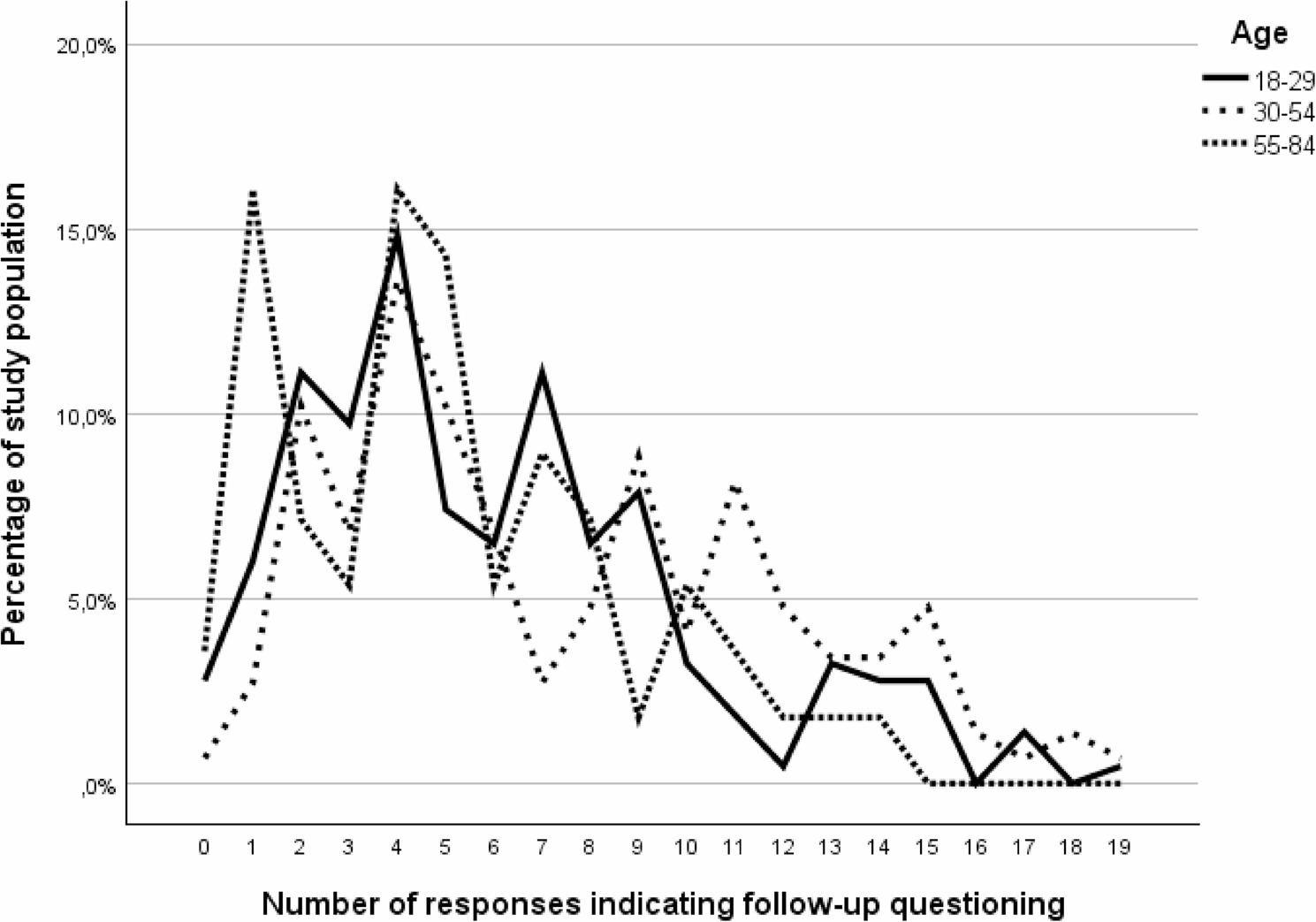
Distribution of the number of responses indicating follow-up questioning relating to age groups

A one-way ANOVA revealed that there was a statistically significant difference in mean responses indicating follow-up questioning between at least two age groups (*p* < 0.001). Tukey’s HSD Test for multiple comparisons found that the mean value for responses indicating follow-up questioning was significantly different between the youngest age group [18–29] and the middle age group [30–54] (*p* = 0.004, 95% C.I =[-2.46,-0.38]), as well as between the oldest age group (55–84) and the middle age group [30–54] (*p* < 0.001, 95% C.I =[-3.88,-0.83]), in that the middle age group had a higher mean value of possible responses indicating follow-up questioning than both other age groups. There was no statistical difference in mean responses indicating follow-up questioning between the youngest age group and the oldest age group (*p* = 0.284, 95% C.I =[-0.52, 2.40]). The distribution of responses requiring follow-up in relation to the age groups is outlined in Fig. 3.

**Fig. 4:**
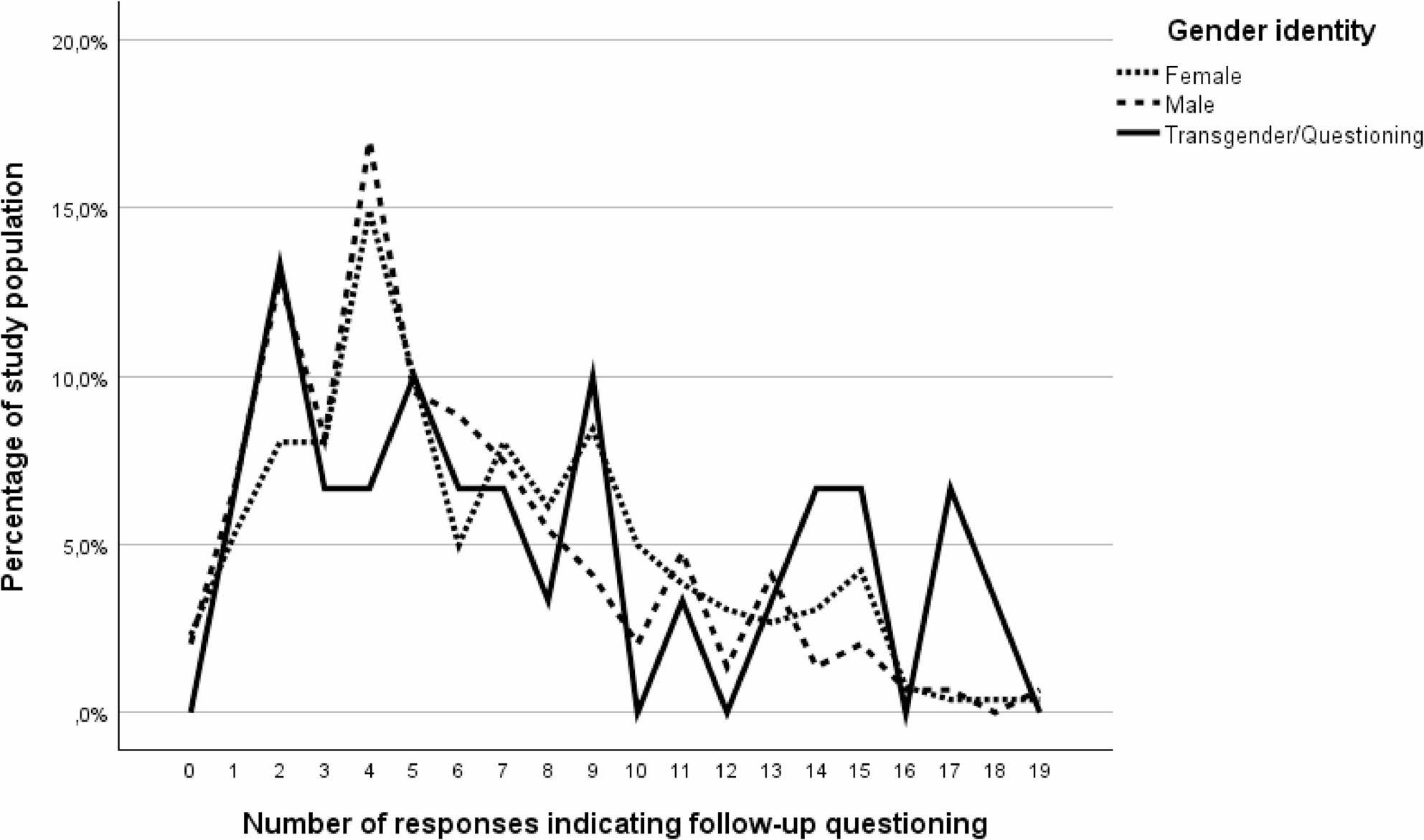
Distribution of the number of responses indicating follow-up questioning relating to gender identity

Furthermore, a one-way ANOVA initially indicated a statistically significant difference in mean responses indicating follow-up questioning between at least two gender identity groups (*p* = 0.034). However, post hoc testing done to investigate between which age groups statistical differences occurred, Tukey’s HSD Test for multiple comparisons, found no significant differences between female and male respondents (*p* = 0.135), female and transgender/questioning respondents (*p* = 0.359), or male and transgender/questioning respondents (*p* = 0.056). The distribution of responses requiring follow-up in relation to gender identity is illustrated in Fig. 4.

**Fig. 5:**
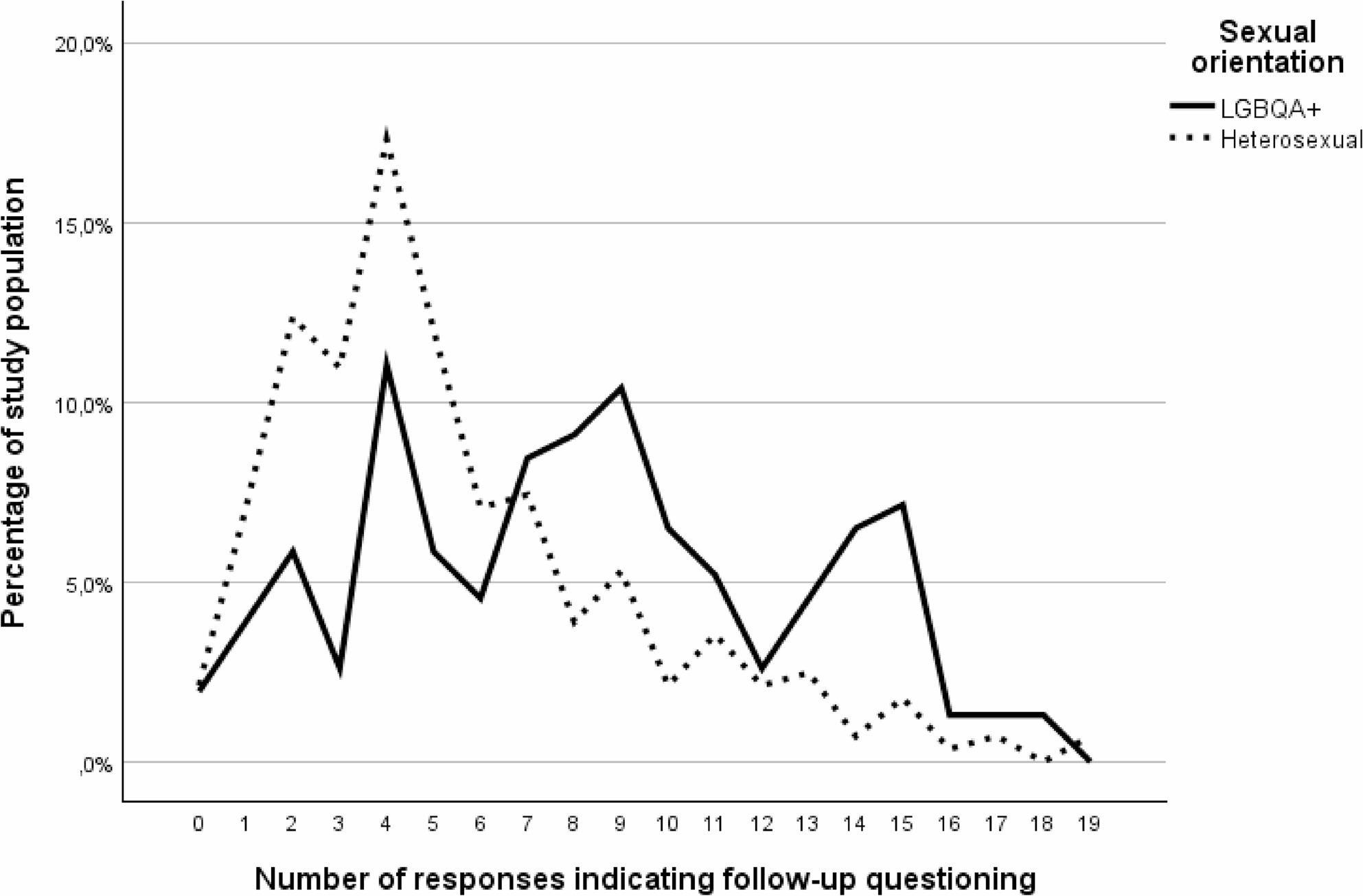
Distribution of the number of responses indicating follow-up questioning relating to sexual orientation

**Fig. 6:**
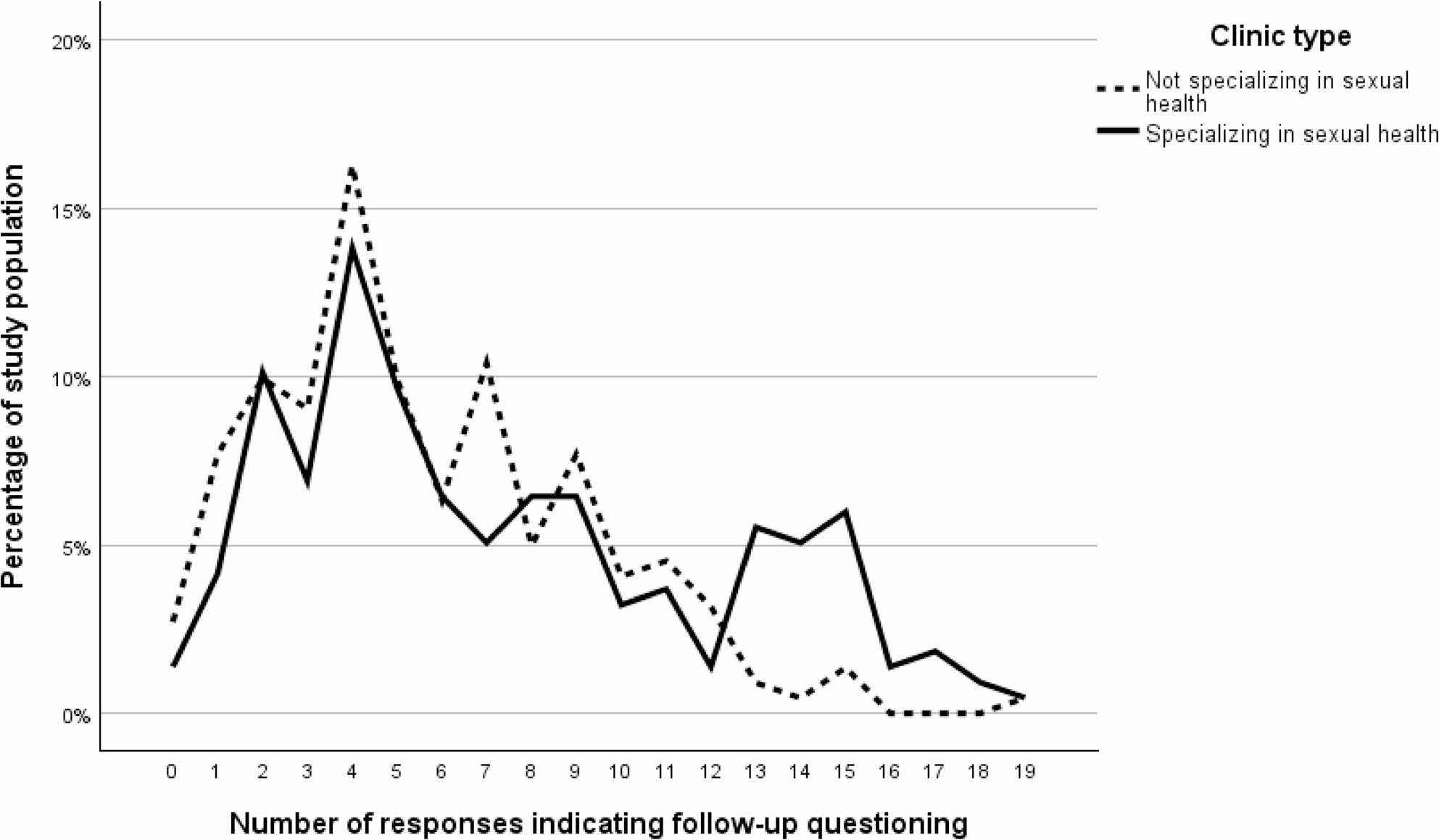
Distribution of the number of responses indicating follow-up questioning between clinic types

As presented in Table 3, response patterns were analyzed with an independent Student t-test for sexual orientation and clinical setting. LGBQA+ participants had significantly higher mean responses indicating follow-up questioning compared to heterosexual participants (*p* < 0.001, 95% C.I =[1.90, 3.57]), as demonstrated in Fig. 5. Furthermore, a difference was observed between the two types of clinics, where sexual health clinic visitors had a significantly (*p* < 0.001, 95% C.I =[-2.47,-0.90]) higher mean value of responses indicating follow-up questioning compared to patients visiting the other clinics. Specifically, there was a difference in that sexual health clinic visitors more frequently gave 13 or more responses indicating follow-up questioning, as seen in Fig. 6.

## Discussion

More than 90% of the respondents in this sample of Swedish outpatients answered the SEXIT Adult questionnaire in a way that, according to the handbook, indicated follow-up questioning from the HCPs. This indicates that there is a great need for in-depth conversations on SRV and suggests that the SEXIT Adult method is effective in identifying when the need for such conversations is indicated. Furthermore, although outpatients consulting clinics specializing in sexual health gave more responses in need of follow-up questions compared to outpatients at the other clinics, the differences were mainly concentrated among patients giving at least 13 or more such responses. The overall high frequency of reported adverse experiences highlights the possible and, as of yet, unmet need of outpatients to have discussions on SRV, as these topics can affect and be affected by both somatic and mental health [7–10, 12]. However, some of the patients’ responses may be misreported, mainly the SEXIT-questions that likely induce fear of negative repercussions, e.g. the questions on giving compensation for sexual acts, perpetration of physical and sexual violence. This risk of misrepresented responses cannot be disregarded, as previous research has suggested that social desirability likely causes underreporting of sexual behaviors considered undesirable and stigmatizing [67].

Sexually diverse respondents stood out as having more responses requiring follow-up than heterosexual respondents. While these results differ from the latest Swedish general population study [68], international studies have shown a higher occurrence of sexual health concerns among sexually diverse individuals compared to heterosexual individuals [69–71]. The differences observed in this study further underline the need for sexual diversity-informed health care [72, 73].

The SEXIT Adult questions helped identify the need for in-depth discussions on SRV regardless of the age of the participants. Notably, the middle age bracket of this study, ages 30–54, stood out compared to both the younger and the older cohorts, regarding SRV concerns. These results contrast with previous research mainly focusing on youth and young adults as “at-risk” populations [42, 44], but may highlight middle-aged populations as being neglected in sexual health and risk-taking research [74]. However, concerning age, there was a skewed distribution of age groups, namely few older outpatients at the clinics specializing in sexual health. Future research is necessary, specifically, to explore the use of the SEXIT Adult method with older outpatients. While sexual activity and sexual satisfaction are increasing in older cohorts, studies also show that sexual health satisfaction often decreases after retirement, when most age-related health concerns become more common (Beckman et al., 2014; Henning et al., 2022; Vasconcelos et al., 2022).

Previous research has shown that both HCPs and outpatients have doubts about the appropriateness of conversations on SRV in outpatient consultations [1–3, 13]. While this study suggests that patients do disclose possible risk and health concerns with the help of the SEXIT Adult questionnaire, it is important that future qualitative studies examine how outpatients and HCPs experience the SEXIT Adult method in the everyday clinical setting of Swedish health care. Future quantitative studies should be performed with larger samples and in specific clinical settings targeting adult outpatients, e.g. SEXIT Adult at a primary care level, and at a psychiatric care level, to explore how the method works in different health care conditions. Additionally, further research is required to examine the usability of the SEXIT Adult method with non-Swedish speakers. If SEXIT Adult is found to be effective, implementation studies will be required to understand how the method can be accepted and employed as a clinical tool.

While further research is needed before SEXIT Adult can be recommended for widespread implementation, the present study highlights a substantial unmet need among Swedish outpatients for healthcare professional–initiated discussions about SRV. These findings suggest that healthcare services and policymakers should consider strengthening routine approaches to identifying and addressing SRV within outpatient care.

### Limitations

There are several limitations that inform the interpretation of the results of this study. Although this study identifies how often patients responded to the SEXIT Adult questionnaire in a way that indicated follow-up questioning, the current study cannot confirm how often follow-up questioning and in-depth conversations between the patient and HCPs occurred.

The total number of questionnaires collected allows for analysis and comparisons between clinics specialized in sexual health and clinics not specialized in sexual health, but comparisons on specific clinic level and between professions were not allowed for. Moreover, there is a risk that the sample of patients consulting the clinics not specialized in sexual health is not representable compared to the other patient sample in the study, given that the six participating clinics not specialized in sexual health were more diverse in which patient groups they targeted than those specialized in sexual health.

As discussed above, another possible limitation of this study is that patients may be underreporting experiences and concerns, mainly due to social desirability and fear of negative repercussions.

The previously mentioned skewness in the sample is another limitation, i.e. respondents who consulted the clinics specializing in sexual health were more likely to belong to the youngest age group, and the oldest age group was small compared to the younger age brackets. Furthermore, only about 7% of the sample (30 individuals) stated that they identified as transgender/questioning; thus, the analysis of their responses needs to be cautious.

An additional limitation is that this study only included Swedish-speaking participants, thus missing out on the experiences of non-Swedish speakers, an overall large group within the Swedish society.

## Conclusion

This study showed that SRV concerns were common among this mixed sample of Swedish outpatients, underlining the benefit of HCP-initiated conversations on these topics. Furthermore, the sexually diverse individuals in this sample stood out in responses indicating follow-up questioning, implying the necessity for sexual diversity informed care. Lastly, the SEXIT Adult method aids HCPs in identifying the need for in-depth conversations on SRV. Future research on both patients’ and HCPs’ perspectives on SEXIT Adult is vital to further evaluate the method.

## Supplementary Information

Supplementary Material 1.

Supplementary Material 2.

Supplementary Material 3.

## Data Availability

The datasets generated and analyzed during the current study are not publicly available due to ongoing analysis within the project but are available from the corresponding author on reasonable request.

## Abbreviations

HCP: Health care professional
SRV: Sexual health, risk–taking behaviors and experiences of violence
GSD: Gender and sexual diversity
SEXIT: Sexual health identification tool
SRHR: Sexual and reproductive health and rights
STI: Sexually transmitted infection
