# Supplementary material for: Identifying sexual health concerns, risk-taking and violence using the newly developed SEXIT Adult tool in a Swedish public outpatient sample
Source: BMC Public Health. 2026 Jul 30;26:2369. doi: 10.1186/s12889-026-28764-5 (PMC13476919; doi:10.1186/s12889-026-28764-5)
Supplement: Supplementary file 1 [file 12889_2026_28764_MOESM1_ESM.pdf]

SEXIT Adult – Conversation about sexual health and experiences of violence

sexit

The purpose of this questionnaire is to enable us to provide better care and support. Your participation is voluntary. You can choose which questions you want to answer and select the responses that apply to you the most.

1. Age .... **years**

2. What is your gender identity?
By gender identity, we mean the gender you identify with.

- ☐ Female
- ☐ Male
- ☐ Binary transgender person
- ☐ Non-binary (with or without transgender experience)
- ☐ Other
- ☐ I don't want to categorize myself
- ☐ I don't know

3. What is your sexual orientation?

- ☐ Bisexual
- ☐ Pansexual
- ☐ Heterosexual
- ☐ Queer
- ☐ Homosexual
- ☐ Asexual
- ☐ Other
- ☐ I don't want to categorize myself
- ☐ I don't know

Now we have some questions about sex. Having sex may, but does not necessarily, mean that you have had vaginal, oral, or anal sex. Sex may involve all kinds of different things, such as masturbating alone or with someone else, making out, caressing someone, or being caressed. This may take place during physical encounters, or by telephone or computer.

4. Are you happy with your sexual health?
Sexual health may, for example, involve sexuality, body and function, general health, pleasure, and satisfaction. You can have good sexual health, either if you have sex with yourself or with others, but also if you do not have sex at all and are satisfied with that.

☐ Yes ☐ No ☐ I don't know

5. Do you have any sexual problems?
Examples of sexual problems: low sex drive, problems with erections, painful sex, problems with orgasms, or anxiety related to sex.

☐ Yes ☐ No ☐ I don't know

6. How often do you and your partners use condoms as protection against sexually transmitted diseases?

☐ Always ☐ Sometimes ☐ Never ☐ I don't know ☐ Not relevant

7. Do you have, or have you had, chlamydia, gonorrhea, syphilis, hepatitis, or HIV?

☐ Yes ☐ No ☐ I don't know ☐ Not relevant

8. How often do you and your partners use birth control?

☐ Always ☐ Sometimes ☐ Never ☐ I don't know ☐ Not relevant

9. Have you or a partner had an unplanned pregnancy?

☐ Yes ☐ No ☐ I don't know ☐ Not relevant

10. Have you ever been subjected to non-consensual sexual acts (sex you didn't want)?

This includes by telephone or computer.
Examples of non-consensual sexual acts: being groped, receiving unsolicited photos or pornographic videos, unwanted advances, or other types of sexual harassment.
Other examples are: someone sharing naked photos of you without your consent or that someone pressures, threatens or forces you to have oral, anal, or vaginal sex or to other sexual acts.

☐ Yes ☐ No ☐ I don't know

11. Have you ever subjected someone else to non-consensual sexual acts (sex they didn't want)?

This includes by telephone or computer.
Examples of non-consensual acts: groping, sending unsolicited photos or pornographic videos, making unwanted advances, or other types of sexual harassment
Other examples are: sharing naked photos of someone without their consent or pressuring, threatening, or forcing someone to have oral, anal, or vaginal sex or to other sexual acts.

☐ Yes ☐ No ☐ I don't know

12. Do you find it difficult to communicate about consent in sexual situations?

For instance, you may find it difficult to determine whether the person you are having or want to have sex with wants to have sex with you. It can also mean that it's difficult for you to tell or show someone that you are unwilling or want to stop when it feels uncomfortable or hurts.

☐ Yes ☐ No ☐ I don't know

13. Have you used sex to handle difficult feelings, or to intentionally harm yourself?

This includes by telephone or computer.
Examples are: using sex as an escape or as self-punishment, or that you seek out sexual situations that are very likely to lead to negative experiences.

☐ Yes ☐ No ☐ I don't know

14. Do you lose control over your sexuality and do things you regret or that worry you?

Examples are: how often you use pornography, sex chat, or meet people for sex, or other sexual behaviors that result in suffering or negative consequences.

☐ Yes ☐ No ☐ I don't know

15. Have you ever received compensation for performing sexual acts?

This includes by telephone or computer.
Examples of compensation: money, alcohol, cigarettes, drugs, housing, food, items, or travel. Sexual acts may take place during physical encounters, but they may also involve sharing naked photos, posing or having sex in front of a webcam.

☐ Yes ☐ No ☐ I don't know

16. Have you ever given someone compensation for sexual acts in Sweden or abroad?

This includes by telephone or computer.
Examples of compensation: money, alcohol, cigarettes, drugs, housing, food, items, or travel. Sexual acts may take place during physical encounters, but they may also involve sharing naked photos, posing or having sex in front of a webcam.

☐ Yes ☐ No ☐ I don't know

17. Have you ever been sexually aroused by something that could be harmful to you or others?

Examples are: arousal in response to children, animals, forced sex, exposing one's genitals, peeping on someone, or other non-consensual situations.

☐ Yes ☐ No ☐ I don't know

sexit

**18. Are you, or is someone close to you, concerned about how much alcohol you drink?**	☐ Yes	☐ No	☐ I don't know
**19. Have you taken drugs during the past year?** Examples are: cannabis, amphetamine, heroin, LSD, GHB/GHL, anabolic steroids, unprescribed narcotic medications or other drugs.	☐ Yes	☐ No	☐ I don't know
**20. Has anyone ever restricted or controlled you?** This may involve your finances, choice of partner, friends, clothing, employment/daily activities, or other violations of your right to privacy. It may also involve someone refusing to help you, or preventing you from getting help with your basic needs.	☐ Yes	☐ No	☐ I don't know
**21. Have you ever restricted or controlled someone else?** This may involve someone's finances, choice of partner, friends, clothing, employment/daily activities, or other violations of his or her right to privacy. It may also involve refusing to help, or preventing someone from getting help with their basic needs.	☐ Yes	☐ No	☐ I don't know
**22. Have you ever been subjected to psychological violence or a threat of violence?** This includes by telephone or computer. Examples of psychological violence: being violated, humiliated, threatened, harassed or bullied, or having property destroyed.	☐ Yes	☐ No	☐ I don't know
**23. Have you ever subjected someone to psychological violence or a threat of violence?** This includes by telephone or computer. Examples of psychological violence: violating, humiliating, threatening, harassing or bullying someone, or destroying their property.	☐ Yes	☐ No	☐ I don't know
**24. Have you ever been subjected to physical violence?** Examples of physical violence: being hit, kicked, shoved, choked, or harmed in some other way.	☐ Yes	☐ No	☐ I don't know
**25. Have you ever subjected someone to physical violence?** Examples of physical violence: hitting, kicking, shoving, choking, or otherwise harming someone.	☐ Yes	☐ No	☐ I don't know
**26. When you were growing up, did you ever see anyone in your family being subjected to psychological, physical, or sexual violence?**	☐ Yes	☐ No	☐ I don't know

Please write down anything else you would like us to talk about:
