# Supplementary material for: Identifying sexual health concerns, risk-taking and violence using the newly developed SEXIT Adult tool in a Swedish public outpatient sample
Source: BMC Public Health. 2026 Jul 30;26:2369. doi: 10.1186/s12889-026-28764-5 (PMC13476919; doi:10.1186/s12889-026-28764-5)
Supplement: Supplementary file 2 [file 12889_2026_28764_MOESM2_ESM.pdf]

SEXIT Adult - Conversation about sexual health and experiences of violence

THE HANDBOOK

SEXIT Adult, version 1.0. Developed by the Knowledge Center for Sexual Health in Region Västra Götaland as part of the research project ”Adaptation and evaluation of SEXIT Adult” (The material is not to be distributed outside of the project).

Contact: sofia.hammarstrom@vgregion.se

Table of Contents

Purpose of SEXIT Adult ... 3

What is SEXIT? ... 4

Why SEXIT is needed ... 5

Working with sexual health ... 7

How should SEXIT be used? ... 8

- Preparations ... 8
- Routines at the clinic ... 9
  - Before the visit ... 9
  - During the visit ... 9
  - After the visit: ... 10
- Research administration ... 10
- Documentation ... 11

Treatment intervention support ... 12

Judicial concerns ... 67

- Confidentiality ... 67
- Suspicion of crime ... 67

References ... 68

Glossary ... 73

Purpose of SEXIT Adult

The purpose of SEXIT Adult is:

to facilitate the initiation of conversations about sexual health, sexual risk-taking and experiences of violence in outpatient consultations,

to, through a concrete method, increase knowledge and confidence among staff to recognize and manage sexual health concerns, sexual risk-taking and experiences of violence in outpatient consultations,

to present a possible structure for asking patients questions about sexual health, sexual risk-taking and experiences of violence in a standardized manner, with the aim of achieving more equitable care

What is SEXIT?

SEXIT is a method consisting of three parts:

Training
Having basic knowledge about sexual health is important for all healthcare professionals who meet patients. Therefore, a training course has been designed within SEXIT with the aim of ensuring that clinics that use SEXIT are well prepared to initiate conversations about, assess and treat sexual health concerns. Through training, participants gain knowledge about different types of sexual health concerns, experiences of violence and risk-taking. Participants in the course can also reflect on and develop their personal treatment of patients from a norm-critical perspective, receive guidance on how different concerns can be handled during the outpatient consultation, and obtain information about other clinics to which patients can be referred for continued help with any health concerns. The participants also learn routines linked to SEXIT. The training course can be given both digitally and physically and contains both lectures and practical exercise elements.

Questionnaire
The SEXIT Adult questionnaire has been developed by a national expert group that has, based on the youth version of the SEXIT questionnaire and using a Delphi methodology, identified questions that are central to conversations with adult patients about sexual health, sexual risk-taking and experiences of violence. A total of 26 questions with fixed response options are included. The questionnaire takes approximately five minutes to answer. In practice, the questionnaire functions as a basis for a conversation during the outpatient consultation, as the patient and healthcare provider jointly review the answers and, if necessary, follow up with further questions. The questionnaire does not contain any personal information that directly links a person to the questionnaire.

Handbook
The SEXIT handbook has been developed as an aid in the daily work on sexual health. The treatment intervention support section contains background information on each question, suggestions for follow-up questions that may be appropriate to ask, and recommended interventions. The SEXIT handbook also contains routines, support for documentation and follow-up, and a detailed reference list for those who want to delve further.

Why SEXIT is needed

- **Because sexual health is part of general health**
- **Because sexual health concerns and experiences of violence are commonly experienced**
- **To better recognize and meet the needs for care and support regarding sexual health and experiences of violence**
- **Because many healthcare professionals lack training in this area and the subject can be experienced as difficult to address**
- **Because patients rarely bring up sexual problems or experiences of violence of their own accord**
- **Because studies show that patients want to be asked about sexual health and experiences of violence**
- **Because there is a lack of evidence-based routines and tools to facilitate conversations about sexual health, sexual risk-taking and experiences of violence**

Sexual health is part of general health; thus, it is important that healthcare professionals ask patients questions about sexuality and sexual health. Several of our common diseases and common health problems affect sexual health, e.g., diabetes, depression, cancer and cardiovascular disease. In Sweden, 8% of the population report that they have had health problems or physical problems that have affected their sex life in the past year. Despite this, only 13% of these respondents report that they have sought advice or help from healthcare professionals for their problems (1). Studies have shown that patients want to be asked questions about this by healthcare professionals, but that they seldom dare to bring up the subject themselves (2).

Healthcare professionals also have an important role in identifying people who are sexually risk-taking or who have experienced psychological, physical or sexual violence. Sexual risk-taking can include not protecting oneself against STIs or unwanted pregnancy, using alcohol or drugs in a way that affects one's judgment, and seeking out situations that involve danger or harm. Sexual risk-taking can lead to health concerns, such as sexually transmitted infections (STIs) and unwanted pregnancies that affect both the individual patient and their partners. People who have witnessed or been exposed to violence are at high risk of several different types of health concerns in both the short and long term (3). During outpatient consultations, by identifying people who are at risk of harming others, there is an opportunity to help the person themselves change their behavior, as well as prevent others from being exposed to criminal acts.

In recent years, Sweden has taken clear steps forward in its work on sexual and reproductive health and rights through implementing a national strategy for sexual and reproductive health and rights (SRHR) and a related national action plan. The documents state that the overall goal is to achieve good, equal and equitable sexual and reproductive health in the entire population, and that the healthcare system has a particularly important role and responsibility in this work (4). However, within the healthcare system itself, this goal is not as clearly acknowledged. Fragmented and inadequate knowledge of the subject area, together with a lack of routines, has led to a situation where questions are rarely raised in outpatient consultations. The explanation for the subject area's absence within healthcare can be found, among other things, in our history and societal view of sexuality as private and surrounded by unwritten rules, norms and taboos.

Unlike many other healthcare fields, where there are rating scales, questionnaires and other methods to support healthcare professionals, there is currently no structured methodology for the assessment and management of sexual health concerns, either in Sweden or in the rest of the world. Therefore, the

development of the SEXIT method has been in demand. Moreover, it is unique, bridging an important gap in both research and clinical practice (5).

A situation where patients are not given the opportunity to address their problems, and where healthcare providers are not equipped to identify sexual health, sexual risk-taking and violence, means that many patients today do not receive the care required for their problems. Health concerns that could be avoided if treated correctly create unnecessary healthcare costs and suffering over time. Without a structured conversation that includes sexual health, sexual risk-taking and experiences of violence, it is difficult to provide effective, person-centered assessment, counseling and treatment to prevent relapse into risk-taking behaviors and future health concerns. SEXIT is a method that can help to resolve this situation of unequal care.

Sexual and Reproductive Health and Rights - SRHR (fact box)

Sexual and reproductive health can be defined as a state of physical, emotional, mental and social well-being in relation to all aspects of sexuality and reproduction, and not merely the absence of disease, dysfunction or injury. Therefore, a positive approach to sexuality and reproduction must consider the role that pleasurable sexual relationships, trust and communication play in self-esteem and general well-being. All people have the right to make decisions about their own bodies and to have access to information and services that support that right.

Achieving sexual and reproductive health requires the recognition of sexual and reproductive rights, which are based on the human rights of everyone to:

• be respected in their bodily integrity, privacy and personal autonomy

• freely define their own sexuality, including sexual orientation, and gender identity and gender expression

• decide whether and when to be sexually active

• choose their sexual partners

• have safe and pleasurable sexual experiences

• decide whether, when and with whom to marry

• decide whether, when and how to have children and how many children they want to have

• have access throughout their lives to the information, resources, services and support necessary to achieve the above, free from discrimination, coercion, exploitation and violence.

(6)

Working with sexual health

The conditions for working with patients vary greatly between different clinics. It is common for healthcare professionals to feel pressured about what can be done during a healthcare visit. Of course, there are many questions that may be important to ask a patient and depending on previous experience and knowledge, it will feel easier to ask about some areas than others.

Sexual health is an area that healthcare professionals often experience as uncomfortable, even though they may understand that it is an important aspect of health. Many healthcare professionals feel intrusive when they ask questions about sexual health. Others feel that they do not have the right skills to deal with the answers they receive from patients. In practice, however, few patients require highly specialized care related to their sexual health. This relationship can be understood using the so-called PLISSIT model developed by Jack Annon (7).

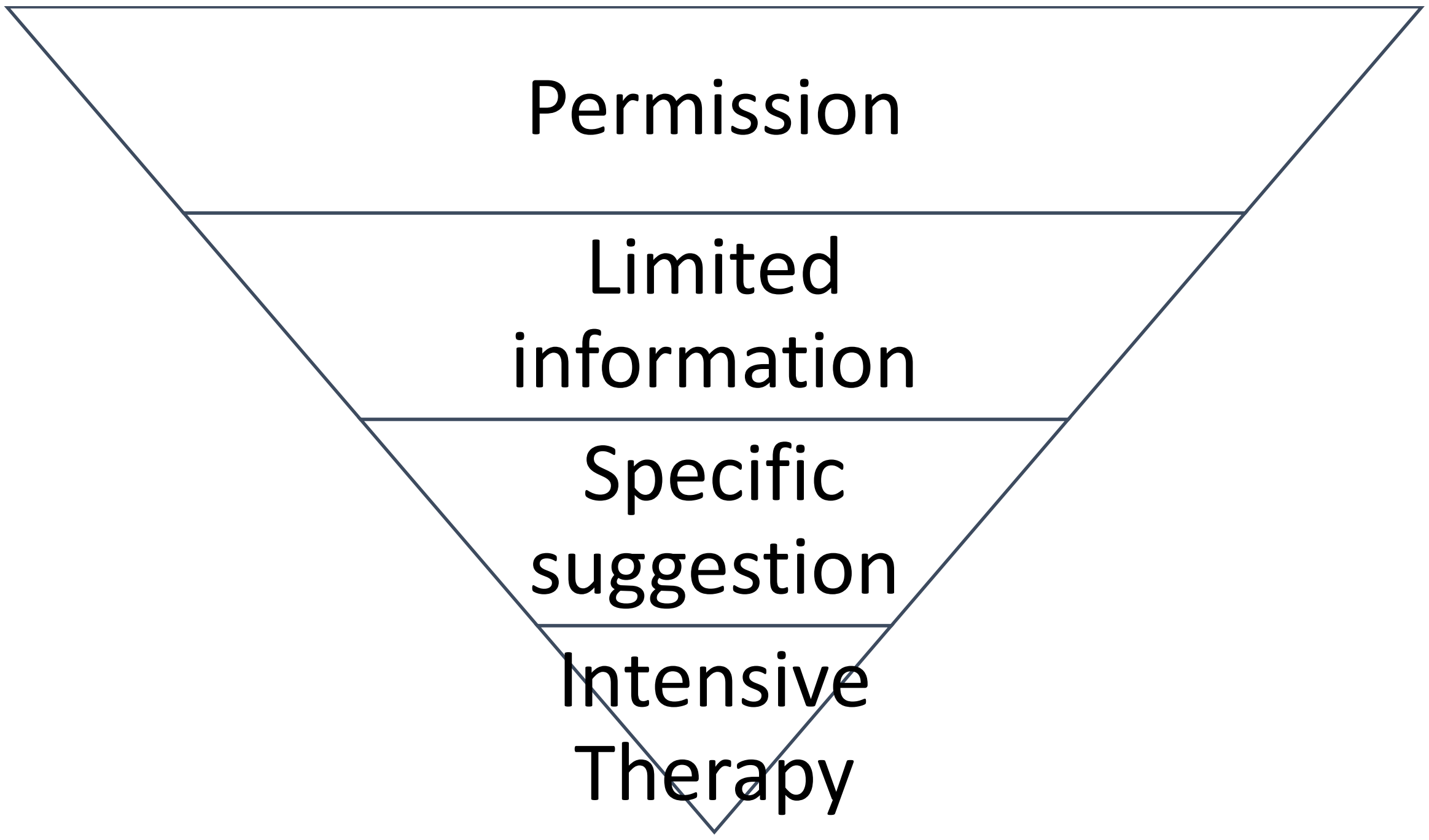

PLISSIT describes what should be offered at the different levels of care related to sexual health and provides a hierarchical flow from general to specialized care. The top of the upside-down triangle above defines the very foundation, that is, what applies to all healthcare clinics. The lower parts show what are considered to apply to clinics with expertise in sexual health.

P stands for "permission". This means that all patients should feel that it is possible to raise questions about their sexual health with their healthcare provider. One way to demonstrate permission can be to ask inviting questions such as **"It is common for people with [health concern] to experience that it affects their sexuality. Do you experience that?"** Even if a patient denies having concerns or does not want to answer the question, the question signals that there is an open climate at the clinic where it is possible to raise these types of concerns, now or later. Other ways of giving permission can be to have accessible materials in the waiting room, such as brochures and posters, which signal that sexual health is an important area that healthcare professionals are prepared to talk about.

LI stands for "limited information". This can involve normalizing and validating what the patient is saying, for example, **"What you have described to me today is true for many people with [health concern] and there are ways to get help for it".** In some cases, healthcare professionals may feel that they do not know what information they can provide. In those cases, it is legitimate to ask for time to find relevant information

and get back in touch with the patient later, for example, **“Thank you for sharing this with me. I will talk to a colleague/my team/consult a specialized clinic, and I will get back to you with an answer on how we can proceed”**.

SS stands for "specific suggestion"; advice or recommendations on how a patient can deal with problems related to their sexual health. Giving specific suggestions requires specific competence in the area, obtained through training and/or appropriate clinical experience. It is always possible to consult colleagues or specialized clinics to determine whether the concerns can be handled in your own clinic or if the patient needs to be referred to another clinic for more specific support.

IT stands for "intensive therapy". This refers to specialized treatment within sexual health and is usually carried out at specialist/expert-level clinics or by healthcare professionals with further expertise, such as sexological training.

Since sexual health has been a neglected area in most Swedish healthcare professionals' education, most healthcare professionals are left to acquire competence in the area themselves. This means there is a risk that some will base their beliefs about what constitutes healthy or unhealthy sexuality on their private experiences and notions when consulting patients. This can lead to both inadequate treatment and incorrect care. It is therefore very important to be aware of the many norms and beliefs that influence how we view both our own and others' sexuality. A good rule of thumb to start from is that there is no such thing as "normal"; there are only norms. By being aware that no one is immune to being influenced by norms and beliefs and by having active discussions about this in the workplace, healthcare professionals can become better at treating their patients in an open and non-judgmental way.

How should SEXIT be used?

Preparations

SEXIT can be used both routinely, for example, during initial consultations, or when in-depth assessment of the topics is required. <u>During this research project, however, SEXIT will be offered systematically, without selecting individual patients</u>.

Regardless of how the clinic has previously worked with sexual health, it is important to be well prepared before introducing SEXIT to the practice. The staff who will use the method must feel confident about why the questions are being asked, which answers indicate a possible risk or health concerns, which follow-up questions should be asked, and which measures are appropriate to take. Uncertainty affects the encounter with the patient and can lead to patients not disclosing any sensitive experiences. For this reason, a specially designed one-day training course has been developed. This manual is a complement to the training course. Previous experiences from SEXIT for adolescents and young adults show that SEXIT works best if the clinic is united, and the staff can consult each other on questions and difficult matters regarding sexual health. It is also good to inventory which local resources and clinics are available for referral, and check that routines that may be needed are in place. This may involve having procedures for how certain issues are handled at the unit, for example, if a patient discloses experiences of having been the victim or admits to having victimized others. **It is therefore recommended that managers and all personnel at the clinic familiarize themselves with the SEXIT method before SEXIT is used. Managers should be prepared for the fact that the introduction of SEXIT may affect other routines at the practice, and it is therefore advisable to provide opportunities for an exchange of experiences and discussion in connection with, for example, workplace meetings**. The introduction of SEXIT is a developmental project and may, therefore, take time.

We also recommend that anyone using SEXIT subscribes to the SEXIT newsletter to stay up to date on any news or updates to the method. The newsletter is published twice a year and can be signed up for through the website www.vgregion.se/SEXIT.

Routines at the clinic

Below are the step-by-step procedures for using SEXIT. During the research project, they have been adapted and supplemented with certain research procedures.

Before the visit

- ✓ **Inform with posters in the waiting room that questions about sexual health, sexual risk-taking and experiences of violence are asked at the clinic within a research project framework.** By using clear information, there will be no surprises when SEXIT is presented, and the patient does not need to feel singled out. The poster informs that the questionnaire is a basis for conversation and that answering it is voluntary. The poster is sent out to the clinics.

- ✓ **Always meet with the patient individually when questions about sexual health, sexual risk-taking and experiences of violence are asked.** If an interpreter is needed, an authorized telephone interpreter is used, if possible, with training in sexual and reproductive health and rights (a so-called "SRHR interpreter"). Relatives are never used for interpretation during conversations based on SEXIT.

During the visit

- ✓ **Distribute the research participant information and provide brief information about the SEXIT research project and why the questions are being asked.**
  Inform patients that the clinic is participating in a research project. Notify them that patients who choose to participate will be asked to complete the SEXIT questionnaire (which is a basis for further conversation), as well as an anonymous survey about how the visit was experienced. The reason for the consultation will be addressed, although some matters that arise may not be possible to discuss during the visit, and may instead be addressed at a follow-up visit. Also, inform patients that participation is voluntary.

  E.g.: "Our clinic is involved in a research project where we offer patients an opportunity to answer a questionnaire with questions about sexual health, sexual risk-taking and experiences of violence because we know that these are important topics that affect general health. The questionnaire is used as a basis for our conversation during your consultation today. Would you consider taking part in the study and filling out the questionnaire? If you want to take part, you will also be asked to answer a short anonymous survey about how you experienced the visit. This is, of course, voluntary, and it will not affect your other care if you choose to refrain". **After the question, the written research participant information and the consent form are always handed out.**

  - o **<u>If the patient wants to participate in the study:</u>** collect a signed consent form. Let the patient keep the research participant information**.**
  - o **<u>If the patient does not want to participate in the study:</u>** Note the patient's gender and age in the attrition form. Continue the visit as usual.

Exclusion criteria. If it is inappropriate to offer participation in the research project, this needs to be noted in the attrition form together with the stated reason for not offering participation. This is generally applicable in the following cases: The patient does not understand the research participant information due to a language barrier or cognitive deficit, or the patient is in acute health distress.

- ✓ **Let the patient answer SEXIT Adult individually.**

- ✓ **Review the answers together.** Even if the patient filled out the questionnaire without indicating any risk or health concern, it is possible that thoughts about sexual health have been awoken and a need to talk about it is experienced. Therefore, make it a habit to always briefly review SEXIT together. Only ask follow-up questions if there is an indication of a risk or health concern.

- ✓ **Always draw attention to a possibly risky habit or negative experience. Use follow-up questions to investigate whether the answer(s) requires action.** An answer that indicates a risk or health concern does not necessarily mean that risk exists or that action is needed. Perhaps a trauma has already been processed or contact with another clinic has already been made. Avoid strong emotional reactions as this may limit the patient's willingness to say more. It is voluntary for the patient to answer questions and follow-up questions.

- ✓ **Make a balanced assessment of the whole based on the visitor's response and the subsequent conversation. Save the SEXIT questionnaire.**

- ✓ **Carry out measures based on the reason for the consultation and assessment of SEXIT.** Deal with the reasons for the consultation, and plan for measures based on what is disclosed through SEXIT. These measures could include, for example, STI testing, referral to a sexual medicine clinic, or more urgent measures such as reporting to Child Protective Services (CPS) or assessing a suspected crime.

- ✓ **Hand over the anonymous patient survey and give instructions.** The anonymous patient survey should be completed by the patient in the waiting room after the visit. Inform them that the survey should be left in the locked mailbox in the waiting room (clearly indicate where it is and make sure the patient has a pen). Thank the patient for participating in the research project.

After the visit:

- ✓ **Document in the medical record that SEXIT has been used, and what is relevant for the patient's further care.**

- ✓ **The completed SEXIT questionnaire and consent form are saved and kept in a secure place.** Keep them in a folder on your desk during the day and put them in a locked cabinet at the end of the day.

Research administration

The research manager will ensure that all documents and materials needed are sent to the clinic. If any materials are running low or if uncertainty arises, the research manager should be contacted as soon as possible (sofia.hammarstrom@vgregion.se).

The following should be available at the clinic:

- Researcher information and consent form
- SEXIT Adult questionnaire and anonymous patient survey
- SEXIT Adult Handbook
- Attrition form

- Pens
- Lockable mailbox in the waiting room
- Posters for waiting rooms and consultation rooms

Empty the mailbox for anonymous patient questionnaires regularly and store these along with the collected SEXIT questionnaires and consent forms in a separate folder, in a locked cabinet in the clinic. It is of great importance that the attrition form is filled out every time someone declines participation or is not asked to participate. The attrition form should be handled carefully and saved for analysis. Those responsible for the research project will provide instructions on how and when the collected material should be sent for analysis.

Documentation

The relevant information that is disclosed through the SEXIT questionnaire and in subsequent conversations based on the SEXIT questionnaire should be documented in the medical record in the same way as information from a consultation when SEXIT is not used. Since SEXIT contains many sensitive questions, it is important to only document what is relevant for continued care and not to summarize unaffected sexual health. Patients should be informed about what will be documented in the medical record or be referred to the digital medical record available via 1177.

By documenting that SEXIT was used during the visit, it becomes easy to know at future consultations what questions were asked and whether it may be appropriate to carry out SEXIT again. It also enables later follow-up on how SEXIT has influenced the choice of care or what measures have been taken at the clinic. To simplify follow-up, documentation at each clinic should be done under the same search term/medical record heading.

Example of phrasings for documentation:

“SEXIT adult has been used during the consultation without need for further action or care”.

“SEXIT adult has been used during the consultation. Experiences of XXX have been disclosed. Follow-up through XXX is ongoing.”

“SEXIT adult has been used during the consultation. Experiences of XXX have been disclosed. Follow-up through XXX is ongoing. A report has been made to Child Protective Services, in accordance with Chapter 14, Section 1 of the Social Services Act.

Treatment intervention support

This section provides a brief explanation of why each question is asked, indications for which answers indicate possible risk or health concern, which follow-up questions are appropriate to ask, and which actions should be considered. The response options have the following indications:

✓ Responses without indication of a health concern or risk-taking

? Responses that indicate a possible health concern or risk-taking

⚠ Answers that clearly indicate a health concern or risk-taking and that require follow-up questions and possibly action

Remember that it is not possible to draw a hard line between the different indications as this is only a guide. Some follow-up questions and actions are the same for several of the questions; if you have already asked the follow-up question, you should, of course, not ask it again. SEXIT is a basis for further conversation, not a screening tool. This means that you, as a healthcare professional, make a balanced assessment of the information from the conversation about SEXIT and from the consultation as a whole.

Difficult cases

When faced with difficult decisions, staff can often find good solutions through dialogue with colleagues and superiors. It is also possible, without revealing the patient's identity, to consult other agencies such as clinics, knowledge centers, municipal organizations, voluntary organizations, local social service offices, prosecutors or the police. We recommend that before using SEXIT, the clinics collect contact information for agencies that may be important to contact in various matters.

Several of the questions in SEXIT can be sensitive to ask and there is a risk that the patient may experience unprocessed emotions, discomfort and/or anxiety. In the event of high anxiety levels or suicidal thoughts, urgent referral for medical assessment at a health center, specialized clinic, psychiatric clinic or emergency room is needed according to local routines.

1. Age:______________

Background

How people relate to their desire and sexuality is not primarily related to age. Access to information and support is needed throughout life to achieve sexual and reproductive health, without the risk of discrimination, coercion or violence.

The ability to feel desire is present throughout life. It is well established that sexual activity in old age strengthens and maintains other health. However, aging may involve physical and psychological changes that affect desire and sexual function. Although a high proportion of older people have an active sex life and are satisfied with their sex life, 47% of women and 23% of men experience one or more sexual problems. When it comes to the sexual health of older transgender people, knowledge is limited (1).

With increasing age, the risk that physiological and mental functions have been affected is greater, but there are good opportunities for medication, aids and sexological advice that can help with, for example, desire, pain problems, erection and lubrication ability.

It is common to hold beliefs that older people do not have sex and that they are not exposed to psychological, physical or sexual violence. These beliefs pose a risk that healthcare providers may overlook patients with health concerns and experiences of violence, so it is important that all patients, regardless of age, are given the opportunity to answer these questions.

Actions

- Consider the patient's age regarding the other questions to identify possible age-related influences, such as menopause, cardiovascular disease, dementia, inflammatory diseases, and osteoarthritis.

2. What is your gender identity?

By gender identity, we mean the gender you identify with. You can specify multiple options.

✓ Female

✓ Male

❓ Binary transgender person

❓ Non-binary (with or without transgender experience)

❓ Other____________

❓ I don't want to categorize myself

❓ I don't know

Background

Research has shown that the proportion of transgender people who have experienced mental and sexual health adversities is greater than within the majority population. A Swedish study of 800 transgender people showed that around a third stated that they had at some point attempted to take their own life (8). It is therefore important that healthcare takes gender identity into account. The question of gender identity provides important information on how the person wants to be addressed and referred to, for example, in medical records. Some transgender people experience suffering because, for example, their body or voice does not match their gender identity, a condition called gender dysphoria. The suffering can make it difficult to undergo physical examinations or to talk about body parts/bodily functions. As healthcare professionals, it is important to check how a patient wants their body/body parts to be referred to and to consider how any physical examination can be carried out in a way that is safe for the patient.

Transgender people have more often experienced psychological, physical and/or sexual violence. Several studies have shown that many have been subjected to abusive treatment or maltreatment, especially linked to their gender expression. Around 40% report that they have been subjected to some kind of sexual violence. Experience of physical violence is more common than for the rest of the population (8, 1). International studies show that trans women are at increased risk of HIV, syphilis and mental illness compared to the general population (9).

For many transgender people, sex with others may trigger gender dysphoria. Nudity can involve a confrontation with bodily and sexual aspects that do not match the gender identity. This is common, especially before and while waiting for gender-affirming care but can also occur after care has been provided. Transgender people may therefore need support in approaching their bodies and sexuality in a way that is positive and affirming.

Gender-affirming hormone therapy, such as testosterone and estrogen, may affect sexual function. For example, this can include fragile/dry mucous membranes or erectile dysfunctions that can make sexual practice difficult. Some people may need treatment such as topical estrogen or potency-enhancing medication.

Follow-up questions

What name and pronoun (for example, he/she/them) would you like me to use?

How do you want me to call different body parts?

Is there anything I can do to facilitate the physical examination?

Is gender or gender identity a topic you have concerns about?

Are you undergoing any gender affirmation treatment?

Are you open about your gender identity with those close to you? What about other contacts? For example, healthcare professionals, colleagues, etc.?

Have you experienced negative treatment in healthcare in relation to your gender identity/gender expression?

Actions

- Counseling and supportive/explorative conversations on gender identity. Explain that each person decides their own identity. Gender identity assessment teams are tasked with trying to determine which people require gender-affirming care such as hormone therapy and surgical procedures. There are many ways to explore gender identity, for example, by using clothing, makeup and other expressions that are in line with one’s identity.
- Inform the patient about the website transformering.se where there is more information about gender identity and transgender care.
- Inform the patient about sexologist clinics/gender identity clinics.
- Consider referral or collaboration with others, such as social services, primary healthcare centers, sexology clinics, or psychiatric services.
- In the event of previous experiences of negative treatment in healthcare, validate the patient's experience and explore what could contribute to a positive experience in the future.

3. What is your sexual orientation?

- Bisexual
- Pansexual
- Heterosexual
- Queer
- Homosexual
- Asexual
- Other___________________________________
- I don't want to categorize myself
- I don't know

Background

Some people find it important to define their sexuality and see it as a central part of their identity, while others do not want or see the need to define themselves.

Human sexuality is on a spectrum and therefore cannot be easily categorized. It is not just about sexual attraction. For example, some people may have experiences of same-sex sexual experiences without defining themselves as gay or bisexual. Whether a person wants to define themselves or share their sexual orientation must always be entirely up to that person.

Despite a wide variation in gender and sexuality, our society is characterized by heteronormativity. This includes beliefs that there are only two genders, that people are only attracted to the opposite sex, that everyone has a desire to have children, and that people should live in monogamy. Heteronormativity also includes an expectation that everyone of reproductive age should feel desire, romantic feelings, and live out their sexuality with themselves and others. All norms become oppressive for those who break them. For people who are asexual, as an example, this may lead to being questioned by partners and caregivers.

As healthcare professionals, it is important to use inclusive and non-normative language when consulting patients, for example, using words like “partner/partners”, and to avoid words like “normal”, instead using words like “usual”. It is always better to ask follow-up questions and ask for clarification when unsure than to interpret what the patient means based on your preconceptions.

Regarding sexual health concerns and experiences of psychological, physical and sexual violence, there is generally an increased incidence among people with non-normative sexuality, such as homosexuals and bisexuals (1). Men who have sex with men (MSM) have a statistically increased risk of sexually transmitted infections such as HIV, STIs and viral hepatitis (9).

Follow-up questions

Is this a question you have concerns about?

Do you have experience of sexual practice in accordance with your sexual orientation?

Have you received the information you feel you need for sexual practice in accordance with your sexual orientation?

Have you experienced negative treatment in healthcare in relation to your sexual orientation? For example, it could be that healthcare professionals assume that you have a different sexual orientation than you do.

Actions

- Counseling and supportive conversations about sexual orientation. For example, explain that there is a wide variation in human sexuality. Just as with gender identity, it is up to each person to define their sexuality and not everyone wants to define themselves.
- Provide recommendations about healthcare clinics, such as sexual health clinics and organizations that offer support around sexual identity, e.g., RFSU and RFSL.
- Consider referral or collaboration with others, such as social services, primary healthcare centers, sexology clinics, or psychiatric services.
- Inform the patient about the possibility for men who have sex with men (MSM) and transgender people who have sex with men to receive hepatitis B vaccination (free of charge) and where testing for sexually transmitted infections can be provided.

4. Are you happy with your sexual health?

Sexual health may, for example, involve sexuality, body and function, general health, pleasure, and satisfaction. You can have good sexual health whether you have sex with yourself, with others, or do not have sex at all and are satisfied with that.

✓ Yes

⚠ No

? I don't know

Background

Most of the Swedish population aged 16–84 are satisfied with their sex life, think sex is important and have had sex in the past year. However, for example, gender identity affects satisfaction, with fewer transgender people being satisfied with their sexual health than cisgender people, 32% versus 57% (1).

What constitutes good sexual health is individual and is not limited to factors such as frequency or the number of partners.

The view on sexuality/sexual health is greatly influenced by different norms. These include norms about who a person is allowed to have sex with based on, for example, age, gender, ethnicity, body and functionality. The norms may also include how many people one is allowed to have sex with, how many people an individual can have a relationship with at the same time, how often an individual should have sex, and how sex should take place, i.e., sexual practices. For example, in our culture, there is a strong norm that sex with other people always involves penetrative/enveloping sex. It is important to be aware that many people enjoy other sexual practices more, such as making out, cybersex, dry sex or oral sex.

Throughout history, people who break norms have been shamed in society. The idea of what is acceptable, nice, or disgusting is constantly conveyed in the media and public debate as well as in break rooms and among friends. Many have heard condescending comments about their sexual practices and expressions, which can make the person shy away from talking about them in a healthcare consultation. In outpatient conversations about sexual health, it is therefore important for the healthcare professional to have a consistently norm-conscious perspective.

Follow-up questions

What are you dissatisfied with regarding your sexual health?

Is there something regarding your sexual health you want help with?

Do you have sex with yourself (masturbation)?

Do you have sex with other people? (Can also occur over the phone/computer, for example)

Can you enjoy sex? (Applies to both masturbation and sex with others)

Actions

- Normalize as much as possible.
- Counseling, for example, exploring how norms around sex may have influenced the patient's view of their sexual health.
- Consider referral or collaboration with others, such as social services, primary healthcare centers, sexology clinics, or psychiatric services.
- Provide information about what support is available in the local area or online, for example, sexology clinics and information sites.

5. Do you have any sexual problems?

Examples of sexual problems: low sex drive, problems with erections, painful sex, problems with orgasms, or anxiety related to sex.

⚠ Yes

✓ No

⚠ I don't know

Background

It is common to experience sexual problems at certain times in life. For example, this may involve reduced libido in connection with depression, erectile dysfunction during periods of high stress, vaginal pain during menopause, or anxiety about having sex with others after experiences of sexual abuse. Many people are not aware of how sexuality is related to general health. Therefore, some people may need help understanding what affects sexual health so that the right changes can be made or the right treatment interventions can be given to achieve good sexual health.

Several commonly used medications have documented side effects that affect sexual health. These include antidepressants (mainly SSRIs/SNRIs), medical treatments for hair loss, antipsychotic medications, certain painkillers, and certain blood pressure medications. The most common side effects are decreased libido, difficulty achieving sexual responses, such as erection and lubrication, and absent or delayed orgasm.

Although the SRHR2017 population survey showed that most people are satisfied with their sexual health, it is also common to experience sexual problems. Women reported, among other things, that they lacked interest, desire, pleasure, lubrication and arousal during sex, that they had felt physical pain during sex, and that they experienced delayed or absent orgasm. Men reported, among other things, erectile dysfunctions, premature ejaculation, that they did not have sex in the ways they wanted, and the lack of a sexual partner or the desire for more sexual partners. Gay and bisexual men and transgender people reported a higher incidence of sexual problems compared to heterosexual and cisgender people (1).

Some sexual problems can be age-related, for example, there is a connection between older age and dry mucous membranes in the vagina, as well as impaired erectile function due to impaired vascular function in the penis.

Follow-up questions

What sexual problems do you experience?

How do these problems affect you?

Is this something you would like help with?

How long have you had the problems?

Do you have any won ideas on whether the problems could be caused by something? Was something else going on in your life when the problems started?

Have you tried anything yourself to lessen the problems?

Have you sought help before? Have you undergone any medical/physical examinations?

Have you received any medical treatment for your symptoms?

Are you taking any other medications?

Are you in/have you gone through menopause? How has it affected you?

Do you have any other health-related conditions (either mental and physical) that may affect your sexual health? Are you receiving treatment for them?

Actions

- Normalize as much as possible. Tell people that sexual health is part of general health and that it is common to experience sexual problems from time to time. At the same time, consider people who have experienced problems over a longer period and who may need care such as medication, aids, physiotherapy, sexological or psychological treatment.
- Provide counseling, for example, explore how norms around sex may have influenced the patient's view of their sexual problems.
- Review any medications that may affect sexual health.
- Consider referral or collaboration with others, such as social services, primary healthcare centers, sexology clinics, or psychiatric services.
- Provide information about what support is available in the local area or online, for example, sexology clinics and information sites.

6. How often do you and your partners use condoms as protection against sexually transmitted diseases?

✓ Always

⚠ Sometimes

⚠ Never

⚠ I don't know

? Not relevant

Background

Condom use in Sweden has increased in recent years, but the increase has occurred primarily in the 16–24 age group, where 68% have used a condom in the past year. Older age groups use condoms to a lesser extent (10). Around 15% of the 16–84 age group have had intercourse without a condom with a casual partner in the past year, which carries a risk of STI (1).

Many adults have received condom information in connection with sex education at school and may not have gone many years without using condoms as protection during sex before requiring condom use again. Adults also have significantly less access to free condoms and condom counseling. It is therefore not surprising that there are misconceptions about condoms and that many patients need condom information. For example, it is common to underestimate how important it is to find the right fit. Most condoms do not differ in length but in width. The condoms available on the market vary between 45–69 mm in width. “Condom measuring tapes” can help provide suggestions for condoms in the correct sizes. Without the right size of condom, the sexual experience may be less pleasurable/enjoyable, and the risk of choosing not to use condoms as protection increases. Condoms also vary in other aspects, such as thickness, material, structure, flavor and other properties, which can make them easier to use and may enhance the pleasure experience for users. Vaginal condoms can be an alternative to condoms, and dental dams can be used for oral sex (11).

Another barrier to condom use may be difficulties in communicating in sexual situations. For example, it may feel awkward to pause a sexual situation and ask about a condom, or there may be a perception that the partners prefer condomless sex (12).

Don't assume that only the person wearing the condom needs information. Everyone, regardless of gender, should be offered condom information.

People in the MSM group (men who have sex with men) and other people at increased risk of HIV transmission should receive information about PrEP (pre-exposure prophylaxis), which is a medication taken for HIV prevention purposes (13).

Follow-up questions

How do you protect yourself when you have sex?

What experiences have you had with using condoms in the past?

In which contexts do you use a condom? In which contexts do you not?

Have you managed to find the right size?

What might increase the likelihood of you using a condom?

Have you received condom information before? What do you remember from it?

Actions

- Condom counseling. Advise on "condom tape measures". There are free versions to print online.
- Inform and counsel on pregnancy testing or emergency contraception within five days (emergency pill or copper coil).
- Contraceptive counseling.
- STI counseling. Inform the patient about the risk of transmission during various sexual practices, including anal and oral sex.
- Conversations about other safer sex practices, such as oral sex and dry sex.
- Information and guidance on testing for sexually transmitted infections. It is a good idea to use the STI sampling template that you can find at https://www.vgregion.se/halsa-och-vard/vardgivarwebben/amnesomraden/kunskapscentrum-for-sexuell-halsa/material-och-publikationer/sti-provtagningsmall/
- If possible, offer the patient condoms from the clinic. Otherwise, advise the patient on where they can find free condoms in their area, such as at STI clinics in connection with testing.
- Inform the patient about the possibility for men who have sex with men (MSM) and transgender people who have sex with men to receive hepatitis B vaccinations (free of charge).
- Decide on a referral for PrEP to an infectious disease clinic or STI clinic.

7. Do you have, or have you had chlamydia, gonorrhea, syphilis, hepatitis or HIV?

⚠ Yes

✓ No

⚠ I don't know

? Not relevant

Background

Previous or ongoing STIs carry an increased risk of new infection (14). Many STIs do not cause symptoms and are therefore difficult to detect without testing. Despite this, only around 10% of people report that they have been tested for any STI in the past year (1). In Sweden, as in the rest of Europe, the incidence of serious STIs such as gonorrhea and syphilis is increasing. There is also currently an increase in antibiotic resistance to treatable STIs such as gonorrhea, which is important to consider when counseling and when STI testing is performed.

Having or having had an STI can be stigmatizing and therefore may be associated with anxiety and shame (15). STIs can greatly affect mental health and affected patients may need both support and counseling to process this. The same may apply to people living with someone who has these STIs.

STI testing is done at several different levels of care, but the most common is at a primary healthcare center, STI/general clinic, youth clinic including the Young Men's Clinic, as well as midwifery clinics and maternity clinics.

Follow-up questions

When was the last time you got tested for STIs?

Are you worried that you might have an STI?

Have you had any STIs? Did you get treatment? When?

Have you had or do you currently have any symptoms that could be caused by an STI?

Have you had unprotected sex abroad? Where and when?

Are you vaccinated against hepatitis B?

Actions

- STI counseling. Inform the patient about the risk of transmission during various sexual practices, including anal and oral sex.
- Conversations about other safer sex practices, such as oral sex and dry sex.
- Condom counseling. Recommend a "condom tape measure." There are free versions to print online.
- If possible, offer the patient condoms from the clinic. Otherwise, advise the patient where they can find free condoms in their area, for example, at an STI clinic in connection with a test.
- Information and guidance on testing for sexually transmitted diseases. It is a good idea to use the STI sampling template that you can find at https://www.vgregion.se/halsa-och-vard/vardgivarwebben/amnesomraden/kunskapscentrum-for-sexuell-halsa/material-och-publikationer/sti-provtagningsmall/
- Inform the patient about the possibility for men who have sex with men (MSM), transgender people who have sex with men, and other risk groups to receive a hepatitis B vaccination (free of charge).
- Decide on a referral for PrEP to an infectious disease clinic or STI clinic.
- Consider referral or collaboration with others, such as a primary healthcare centers, sexology clinics, or midwifery clinics.

8. How often do you and your partners use birth control?

✓ Always

⚠ Sometimes

⚠ Never

⚠ I don't know

? Not relevant

Background

Many people may need help choosing an effective contraceptive. Long-acting contraceptive methods such as an IUD or a contraceptive implant should be recommended as they are the most effective in reducing the risk of unplanned pregnancy (16). These methods are also better suited to people who have difficulty remembering to take their medications regularly.

Within five days of unprotected vaginal intercourse, emergency contraception should be recommended to prevent unwanted pregnancy. This can be done with emergency contraception pills or with a copper coil. The copper coil is the most effective method but can be difficult to access. It is important to know that emergency contraception pills have no protective effect after ovulation, which makes the copper coil the only option within this time frame.

Emergency contraceptive pills containing Levonorgestrel should be recommended if the patient has forgotten to take their regular contraceptive pills. One week after using emergency contraceptive pills, barrier protection is recommended during vaginal sex, and a pregnancy test is recommended three weeks after treatment with emergency contraceptive pills.

Many people rely on the person who can get pregnant to use hormonal contraception. Therefore, they neglect the use of other protection, which poses an additional risk of unwanted pregnancy. When consulting patients, remember that some people have a desire for pregnancy and therefore do not protect themselves, and also that there are sexual practices that cannot lead to pregnancy.

For people who have gone through menopause, birth control is not an option; 12 months after the last period, it is safe to stop using birth control. However, people who receive estrogen treatment after menopause may need to have a hormonal coil to protect the uterine lining.

Follow-up questions

What contraceptive are you using now?

How is it working out for you?

Have you considered changing your contraceptive method?

Are you worried about pregnancy?

Do you sometimes forget to take your birth control pills?

Are you aware of the differences between how effective various contraceptives are in preventing pregnancy?

Actions

- Contraceptive counseling.
- Pregnancy test or emergency contraception method within five days (emergency pill or copper coil).
- Information and guidance on testing for sexually transmitted infections. It is a good idea to use the STI sampling template that you can find at https://www.vgregion.se/halsa-och-vard/vardgivarwebben/amnesomraden/kunskapscentrum-for-sexuell-halsa/material-och-publikationer/sti-provtagningsmall/
- STI counseling. Inform the patient about the risk of transmission during various sexual practices, including anal and oral sex.
- Conversations about other safer sex practices, such as oral sex and dry sex.
- Condom counseling. Recommend a "condom tape measure." There are free versions to print online.
- If possible, offer the patient condoms from the clinic. Otherwise, advise the patient where they can find free condoms in their area, for example, at an STI clinic in connection with a test.
- Consider referral to, for example, a midwifery clinic or a gynecological clinic.

9. Have you or a partner had an unplanned pregnancy?

Yes

No

I don't know

Not relevant

Background

People who have previously had an abortion are at increased risk of recurring unplanned pregnancies. Of people who have had an abortion, 46% have previously experienced one or more abortions (17). There are several different reasons why people cannot find a contraceptive that works well for them. For example, fear of hormones, concerns about negative side effects when using them, and lack of knowledge about the effectiveness of different contraceptives can affect the choice of contraceptive method.

An example of a lack of knowledge could be the belief that all condoms are the same size, which leads to the risk of people using condoms that are either too tight or loose, and which therefore can tear or fall off (11). Another explanation is limited access to IUD insertion in connection with abortion and follow-up when contraceptives do not work.

For people with previous abortion experience, long-acting contraceptive methods should be recommended first, as these prevent unplanned pregnancy most effectively (16).

Follow-up questions

How many times have you/your partner been pregnant?

Did the pregnancy continue?

Are you worried about pregnancy?

Have you used emergency contraceptive pills? Repeated emergency contraceptive pills?

Do you have effective contraception today?

Are you aware of the differences in how effective various contraceptive methods are in preventing unwanted pregnancy?

Measures

- Decide whether a pregnancy test (U-HCG) should be taken and whether emergency contraception (emergency contraceptive pill or copper coil) should be given within five days of unprotected sex.
- Contraceptive counseling with a focus on long-term protection.
- Condom counseling. Recommend a "condom tape measure." There are free versions to print online.
- If possible, offer the patient condoms from the clinic. Otherwise, advise the patient where they can find free condoms in their area, for example, at an STI clinic in connection with a test.
- STI counseling. Inform the patient about the risk of transmission during various sexual practices, including anal and oral sex.
- Information and guidance on testing for sexually transmitted diseases. It is a good idea to use the STI sampling template that you can find at https://www.vgregion.se/halsa-och-vard/vardgivarwebben/amnesomraden/kunskapscentrum-for-sexuell-halsa/material-och-publikationer/sti-provtagningsmall/
- Conversations about other safer sex practices, such as oral sex and dry sex.
- Consider referral or collaboration with others, such as social services, primary healthcare centers, sexology clinics, or psychiatric services.

10. Have you ever been subjected to non-consensual sexual acts (sex you didn’t want)?

This includes by telephone or computer. Examples of non-consensual sexual acts: being groped, receiving unsolicited photos or pornographic videos, unwanted advances, or other types of sexual harassment. Other examples are: someone sharing naked photos of you without your consent or that someone pressures, threatens or forces you to have oral, anal, or vaginal sex or to other sexual acts.

⚠ Yes

✓ No

⚠ I don't know

Background

People who have been subjected to non-consensual sexual acts are affected differently. It is not possible to predict how a person will be affected based on the type of act they have been subjected to. There are many norms that lead to beliefs about what “counts” as harassment/abuse. It is important to be aware of your own beliefs about such norms to provide the right treatment of a person who talks about being subjected to abuse.

People who have been subjected to sexual violence show impaired physical and mental health (3). In total, 10% of men and 40% of women in Sweden state that they have experienced some form of non-consensual sexual act. LGBTQ individuals are exposed to a greater extent than others (1). Usually, the perpetrator is someone the victim already knows (18).

Very few people, around 7% of women and 2% of men, seek healthcare after being subjected to any type of sexual violence. Among LGBTQ people, the tendency to seek care varies between 6% and 18% (1).

Getting a physical response to stimulation, such as an erection or lubrication, often occurs in abusive situations. This does not mean that the person was turned on by the situation; it is a completely automatic physical reaction that can be very confusing for the victim. This type of information can be helpful to convey in conversations with someone who has been abused to normalize their possible reactions.

A person who has been sexually assaulted may feel guilt and shame about what happened. It is of the utmost importance to validate the patient and relieve these feelings. You may be the first person the patient has disclosed this information to.

Follow-up questions

What happened? When did it happen?

Has it happened several times?

Is there any reason to believe it could happen again?

How do you feel the events have affected you?

Have you told anyone about what happened?

Have you filed a police report (and secured DNA evidence)?

Have you previously sought help or treatment for this?

Is this something you would like support with?

Actions

- Inform the patient that these actions are criminal unless there was full consent in all cases.
- If less than ten days have passed since the assault, arrange for a forensic examination with the securing of DNA evidence, even if a police report is not currently being considered by the patient (local procedures may apply).
- Pregnancy test and/or emergency contraception method within five days (emergency pill or copper coil).
- Information and guidance on testing for sexually transmitted infections. It is a good idea to use the STI sampling template that you can find at https://www.vgregion.se/halsa-och-vard/vardgivarwebben/amnesomraden/kunskapscentrum-for-sexuell-halsa/material-och-publikationer/sti-provtagningsmall/
- Provide information about hotlines, organizations or other agencies that offer support in the event of sexual assault, such as Utväg, Kriscentrum, Terrafem, Stödlinjen för män, Stödlinjen för transpersonen and Kvinnofridslinjen [Swedish help organizations for survivors of sexual abuse].
- Normalize trauma-related symptoms, explain that the symptoms are a healthy reaction to an unhealthy situation. This helps the patient place the blame on the perpetrator.
- Consider referral or collaboration with others, such as social services, primary healthcare centers, sexology clinics, or psychiatric services.
- If you suspect that a child under the age of 18 is being harmed, file a report to Child Protective Services. Reporting concerns to CPS can also be considered in cases of previous victimization. If necessary, you can inform other relevant parties in the clinic that the report has been made. In the case of domestic violence, do not inform guardians or other relatives about the report. If there is uncertainty about reporting, it is always possible to consult the social services first. Guardians should, as far as possible, be informed that a report is being made.
- If you suspect a crime, consider filing a police report. See page 66 for more information about when confidentiality can be lifted in the event of a suspected crime.

11. Have you ever subjected someone else to non-consensual sexual acts (sex they didn’t want)?

This includes by telephone or computer. Examples of non-consensual acts: groping, sending unsolicited photos or pornographic videos, making unwanted advances, or other types of sexual harassment. Other examples are: sharing naked photos of someone without their consent or pressuring, threatening, or forcing someone to have oral, anal, or vaginal sex or to other sexual acts.

⚠ Yes

✓ No

⚠ I don't know

Background

The vast majority of individuals (about 99%) convicted of sexual offenses are men (19). However, there are also female perpetrators and studies show that the incidence is underreported (20). It is important for healthcare professionals to be aware of this and not to assume who might subject others to non-consensual sexual acts.

Since the issue is surrounded by taboos and far from all crimes are reported or lead to convictions, there are many who do not receive help with their impulses and behaviors of subjecting others to non-consensual sexual acts. There are also individuals who have not yet committed criminal acts and who are in great need of preventive measures from healthcare services. For these individuals, it is extremely important to be asked the types of questions that are included in SEXIT, and to be treated in a respectful manner and offered care (21). There are units with special knowledge in this area, such as PrevenTell. PrevenTell is a national hotline that targets individuals who are worried about losing control of their sexuality and about harming themselves or others. Patients can call PrevenTell themselves, but it is also possible for relatives/next of kin and professionals to consult PrevenTell. PrevenTell is run by ANOVA in Stockholm. ANOVA is assigned to assess and treat individuals with unwanted sexual arousal patterns/paraphilic sexual interests.

Compulsive sexual behavior, or excessive preoccupation with sex, combined with paraphilic interests, such as experiencing attraction toward non-consensual sexual acts, increases the risk of engaging in criminal or harmful acts (22). There is a stigma surrounding paraphilic interests and many patients may find it easier to talk about compulsive sexuality/preoccupation with sex. This may therefore serve as an entry point for the patient to later disclose concerns about their sexual attraction patterns.

Follow-up questions

What happened?

How do you think and do to make sure that sex is consensual?

Have you ever pressured someone into having sex?

Do you feel like you are losing control over your sexuality?

Do you watch porn? How much time do you spend on it?

What kind of porn do you watch?

Have you watched and/or distributed illegal material?

Have you previously sought help or treatment for this?

Is this something you would like support with?

Actions

- Consider referral or collaboration with others, such as social services, a primary healthcare center, a sexology clinic, psychiatric services.
- Information and guidance on testing for sexually transmitted diseases. It is a good idea to use the STI sampling template that you can find at https://www.vgregion.se/halsa-och-vard/vardgivarwebben/amnesomraden/kunskapscentrum-for-sexuell-halsa/material-och-publikationer/sti-provtagningsmall/
- Provide information about, for example, PrevenTell, väljattsluta.se or other agencies that offer support regarding unwanted sexuality and/or violent behavior.
- If you suspect that a child under the age of 18 is being harmed, file a report to Child Protective Services. Reporting concerns to CPS can also be considered in cases of previous victimization. If necessary, you can inform other relevant parties in the clinic that the report has been made. In the case of domestic violence, do not inform guardians or other relatives about the report. If there is uncertainty about reporting, it is always possible to consult the social services first. Guardians should, as far as possible, be informed that a report is being made.
- If there is indication of non-consensual sex – inform the patient that these acts are criminal. Also confirm the positive aspect of the patient disclosing their experiences, which indicates a willingness to change.
- If you suspect a crime, consider filing a police report. See page 66 for more information about when confidentiality can be lifted in the event of a suspected crime.

12. Do you find it difficult to communicate about consent in sexual situations?

For instance, you may find it difficult to determine whether the person you are having or want to have sex with wants to have sex with you. It can also mean that it's difficult for you to tell or show someone that you are unwilling or want to stop when it feels uncomfortable or hurts.

⚠ Yes

✔ No

⚠ I don't know

Background

Free will and consent are prerequisites for good sexual health. Sexual consent can be complex and is perceived differently in different contexts. It can be understood as a combination of an inner feeling of willingness and actions or behaviors that demonstrate that willingness. It is rarely as simple as just saying yes or no to having sex or a specific form of sex. Sexual consent is often expressed non-verbally. A Swedish survey study with over 12,000 respondents aged 18-64 years shows that most people have a good ability to communicate their own consent and to interpret the consent communication of others. At the same time, 63% of women and 34% of men state that they have agreed to unwanted sex (23).

Sexual identity and gender identity also affect the experience of sexual empowerment and sexual consent communication. Bisexual women are more likely than other women to have agreed to unwanted sex. Similarly, gay and bisexual men are more likely than other men to have agreed to unwanted sex (23). In the SRHR2017 population study, individuals with transgender experiences were more likely than cisgender individuals to report rarely or never being able to say no to sex, and to a lesser extent being able to speak up or protest during sex (1).

A majority of the Swedish population feels confident in their ability to interpret others' consent communication; 61% state that they are usually sure that they understand whether a person wants to have sex with them. Six percent often find it difficult to understand whether a person wants to have sex with them and state that they become insecure. A third of all respondents believe that they would benefit from getting help to improve their ability to communicate about sex, but a majority (77%) do not know where to turn for help with these kinds of questions (23).

Follow-up questions

Is this a question you have concerns about?

When do you find yourself unsure whether people you have sex with want it? How have you dealt with that?

When do you find it difficult to express or show that you do not want sex? How have you dealt with that?

Do you find that people you have sex with have difficulty interpreting your signals, for example, when you don't want sex or when it hurts?

Have you had sex with someone even though you didn't really want to? When? Has it happened multiple times?

Have you had sex with someone who you felt didn't really want to have sex with you? For example, have you pressured someone into having sex?

Have you ever reflected on the fact that the sex you had with someone wasn't consensual or mutual?

Have you previously sought help or treatment for this?

Is this something you would like support with?

Actions

- Provide information about consent, that it is not just about saying yes or no but about a whole interaction, including body language, that it can be withdrawn at any time and that just because something felt good or okay to do before does not mean that the person wants to do the same things again. Use metaphors for consent communication, such as the “traffic lights” - green light for what feels good, yellow for what is unsafe and red for what does not feel good.
- Consider referral or collaboration with others, such as social services, a primary healthcare center, a sexology clinic, or psychiatric services.
- Provide information about, for example, PrevenTell, väljattsluta.se or other agencies that offer support regarding unwanted sexuality and/or violent behavior.
- If you suspect that a child under the age of 18 is being harmed, file a report to Child Protective Services. Reporting concerns to CPS can also be considered in cases of previous victimization. If necessary, you can inform other relevant parties in the clinic that the report has been made. In the case of domestic violence, do not inform guardians or other relatives about the report. If there is uncertainty about reporting, it is always possible to consult the social services first. Guardians should, as far as possible, be informed that a report is being made.
- If you suspect a crime, consider filing a police report. See page 66 for more information about when confidentiality can be lifted in the event of a suspected crime.

13. Have you used sex to handle difficult feelings, or to intentionally harm yourself?

This includes by telephone or computer. Examples are: using sex as an escape or as self-punishment, or that you seek out sexual situations that are very likely to lead to negative experiences.

⚠ Yes

✓ No

⚠ I don't know

Background

Sex can affect or change emotional states. Many regulate their emotions through sex, for example, using sex as a pastime, to seek excitement, or to relax when feeling stressed. For most people, such behaviors do not lead to suffering or other negative consequences. Others find that they get stuck using sex as their only or primary way to regulate emotions. These people may need help learning other strategies for managing emotions and solving problems.

There are people who practice BDSM, or who enjoy acts that could be perceived as harmful by others.

Self-harm through sex can involve seeking out sexual situations that can cause physical or psychological harm. The behavior can serve the same function as non-suicidal self-harm, that is, it is dealing with difficult feelings or events. Self-harm through sex can manifest itself in many ways, such as having sex with a person one is not interested in or attracted to, or seeking out situations that involve physical or sexual violence, humiliation, sexual risk-taking, destructive relationships, or transactional sex (24). For some, it can be difficult to define for themselves whether a behavior is positive or not, and therefore it is important, as a healthcare professional, to explore with the patient in a non-judgmental way how they are affected by their behaviors. In some cases, the patient may experience mixed emotions, that is, there are both pleasant and unpleasant feelings linked to the behavior.

Self-harm through sex can be linked to previous sexual abuse and poor mental health. It is therefore common to have previously been in contact with healthcare, for example, for depression, anxiety, eating disorders and suicide attempts (24). Depending on the situation and the person, sex as self-harm can be a sex crime matter.

Follow-up questions

Have your behaviors around sex led to negative consequences for you or others? In what way?

What feelings or situations do you find yourself dealing with through sex?

What sexual behaviors do you engage in?

How do you feel you have been affected when using sex to deal with difficult emotions?

In what ways have you used sex as self-harm?

Has it happened several times? How often?

Who/whom are you meeting?

How do you get in touch with people you meet?

Do you have sex in such a way that there is a risk of serious physical harm?

What are your thoughts about this behavior? What do you think might be causing it?

Have you experienced anything earlier in your life that reminds you of what happens when you hurt yourself with sex?

Have you previously sought help or treatment for this?

Is this something you would like support with?

Actions

- Explore whether the behavior is harmful to the patient or others. If not, normalize as much as possible. Explain that there is wide diversity in sexuality and sexual behaviors.
- Consider referral or collaboration with others, such as social services, a primary healthcare center, a sexology clinic, or psychiatric services.
- Provide information about, for example, Kriscentrum, Stödlinjen för män, Stödlinjen för transpersoner and PrevenTell.se or other agencies that offer support.
- Inform the patient that these actions are criminal if there was no consent to all the actions.
- Conversations about other safer sex practices, such as oral sex and dry sex.
- If you suspect that a child under the age of 18 is being harmed, file a report to Child Protective Services. Reporting concerns to CPS can also be considered in cases of previous victimization. If necessary, you can inform other relevant parties in the clinic that the report has been made. In the case of domestic violence, do not inform guardians or other relatives about the report. If there is uncertainty about reporting, it is always possible to consult the social services first. Guardians should, as far as possible, be informed that a report is being made.
- If you suspect a crime, consider filing a police report. See page 66 for more information about when confidentiality can be lifted in the event of a suspected crime.

14. Do you lose control over your sexuality and do things you regret or that worry you?

Examples are: how often you use pornography, sex chat, or meet people for sex, or other sexual behaviors that result in suffering or negative consequences.

⚠ Yes

✓ No

⚠ I don't know

Background

Some people feel that sex takes up too much of their lives and negatively affects their studies, work, leisure time and/or relationships. These people may feel that they have no control over their sexuality or sexual behavior. Some use sex to regulate anxiety, emotions or moods. In some cases, it can be compared to self-harming behaviors.

It is not uncommon for people to worry about, for example, their pornography consumption, masturbation or other sexual behaviors, but most do not have problems at a level that is considered to require professional care. Often, it is helpful to understand that there is a great variation in how we humans experience sexual desire and how we live out our sexuality. For example, in society there is a strong mononormativity—the assumption that everyone should strive to live in monogamous relationships— which not all people experience as desirable or functional. Such questions can be important to clarify with patients before their sexual behaviors are problematized.

Compulsive sexual behavior or compulsive sexual behavior disorder is a clinical condition where a person has major problems and engages in problematic sexual behaviors such as hours-long sex chats, watching pornography or seeking out sexual contacts. The condition causes suffering and/or negative consequences such as increased stress and anxiety. This leads to neglect of other necessary activities, such as work/education, due to the time spent on sexual behaviors, relationship problems due to acts that involve infidelity, or sleep disturbance due to sexual behaviors that are carried out when the person should be sleeping. It is currently difficult to determine the extent of this type of disorder as there is no consensus on diagnostic criteria yet. However, the majority of those receiving treatment are men, but women are probably underreported (25).

There is evidence-based treatment for compulsive sexuality and preoccupation with sex. Psychological treatment, such as cognitive behavioral therapy with a focus on reducing risk factors for acting out unwanted sexual impulses, is effective. Similarly, libido-suppressing medications such as SSRIs and hormone-modulating medications are also helpful.

Compulsive sexual behavior combined with paraphilic interests (sexual attraction toward non-consensual acts) leads to an increased risk of engaging in criminal or harmful acts (22).

People may use terms such as *sex addiction* or *porn addiction* to describe their own behavior. Although there are some similarities with substance addiction, the differences outweigh the similarities, thus, the condition is not classified as an addiction in standard diagnostic manuals.

Follow-up questions

In what ways do you feel you are losing control over your sexuality?

Do you think this is a problem for you personally, or do you feel it is mainly a problem for those around you?

What consequences does the behavior lead to? For yourself? For others?

How much time do you spend per day on sexual thoughts and behaviors, such as fantasizing about sex, watching porn, having sex with yourself or others?

Do you watch porn? How much?

What kind of porn do you watch?

Have you watched and/or distributed illegal material?

Do you sex chat? How much?

Do you have casual sexual contacts? How often?

Have you previously sought help or treatment for this?

Is this something you would like support with?

Actions

- Explore whether the behavior is harmful to the patient or others; if not, normalize as much as possible. Explain that there is a wide diversity when it comes to sexuality and sexual behaviors.
- Provide information about PrevenTell.se or other agencies that offer support regarding unwanted sexuality.
- Consider referral or collaboration with others, such as social services, primary healthcare centers, sexology clinics, or psychiatric services.
- Information and guidance on testing for sexually transmitted diseases. It is a good idea to use the STI sampling template that you can find at https://www.vgregion.se/halsa-och-vard/vardgivarwebben/amnesomraden/kunskapscentrum-for-sexuell-halsa/material-och-publikationer/sti-provtagningsmall/
- STI counseling. Inform the patient about the risk of transmission during various sexual practices, including anal and oral sex.
- Pregnancy test or emergency contraception method within five days (emergency contraceptive pill or copper coil).
- Contraceptive counseling.
- Condom counseling. Recommend a “condom tape measure.” There are free versions to print online.
- Conversations about other safer sex practices, such as oral sex and dry sex.
- Inform the patient that the person may have been a victim of sexual assault.
- If you suspect that a child under the age of 18 is being harmed, file a report to Child Protective Services. Reporting concerns to CPS can also be considered in cases of previous

victimization. If necessary, you can inform other relevant parties in the clinic that the report has been made. In the case of domestic violence, do not inform guardians or other relatives about the report. If there is uncertainty about reporting, it is always possible to consult the social services first. Guardians should, as far as possible, be informed that a report is being made.

- If you suspect a crime, consider filing a police report. See page 66 for more information about when confidentiality can be lifted in the event of a suspected crime.

15. Have you ever received compensation for performing sexual acts?

This includes by telephone or computer. Examples of compensation: money, alcohol, cigarettes, drugs, housing, food, items, or travel. Sexual acts may take place during physical encounters, but they may also involve sharing naked photos, posing or having sex in front of a webcam.

⚠ Yes

✓ No

⚠ I don't know

Background

Approximately 1.5% of women, 1% of men, and 7% of LGBTQ individuals have experienced receiving compensation for sex (1). Among people aged 16-29, 2% of men and 3% of women have at some point received compensation for sex (26).

There are emotional and material reasons why a person has transactional sex. It can also be a case of coercion. Emotional reasons can, for example, be related to psychological distress or mental health concerns where the symptoms of anxiety are suppressed or are part of self-harming behavior. Material reasons can be about getting money, housing or drugs. People can also be forced into having transactional sex as a form of human trafficking, or it can happen as a result of blackmail, bullying or manipulation.

There may also be positive motivations for the person, such as the feeling that it is fun and exciting (27, 28). Regardless of the underlying motive, people with experiences of transactional sex are more likely than others to have experienced sexual abuse, and many have experienced physical and psychological violence (29).

Sex for compensation can take many forms and can be performed in different ways, for example, street prostitution, massage parlors, escort services and “sugar dating”, but it can also take place in the form of pictures and via video calls (so-called “cam shows”) sold on the internet, porn or strip clubs, and pornographic film production. Contacts between sellers and buyers often take place via the internet.

In Sweden, it is not illegal to receive compensation for sexual acts, but it is a criminal offense to provide compensation for sexual services.

Follow-up questions

What happened?

Have you ever considered seeking compensation for sexual acts?

Have you been offered compensation for sex?

Have you told anyone about it?

Have you thought about this afterwards?

How did it happen that you got compensation for sex?

How do you feel this affects you?

Have you previously sought help or treatment for this?

Is this something you would like support with?

Actions

- Inform the patient that it is not illegal to receive compensation for sex, but that it is illegal to give compensation.
- Pregnancy test or emergency contraception method within five days (emergency contraceptive pill or copper coil).
- Information and guidance on testing for sexually transmitted diseases. It is a good idea to use the STI sampling template that you can find at https://www.vgregion.se/halsa-och-vard/vardgivarwebben/amnesomraden/kunskapscentrum-for-sexuell-halsa/material-och-publikationer/sti-provtagningsmall/
- STI counseling. Inform the patient about the risk of transmission during various sexual practices, including anal and oral sex.
- In the event of abuse: If <10 days have passed since the abuse, arrange for a forensic examination with DNA evidence, even if a police report is not currently in question for the patient (local procedures may apply).
- Contraceptive counseling.
- Condom counseling. Recommend a "condom tape measure." There are free versions to print online.
- Conversations about other safer sex practices, such as oral sex and dry sex.
- Consider referral or collaboration with others, such as social services, primary healthcare centers, sexology clinics, or psychiatric services.
- If you suspect that a child under the age of 18 is being harmed, file a report to Child Protective Services. Reporting concerns to CPS can also be considered in cases of previous victimization. If necessary, you can inform other relevant parties in the clinic that the report has been made. In the case of domestic violence, do not inform guardians or other relatives about the report. If there is uncertainty about reporting, it is always possible to consult the social services first. Guardians should, as far as possible, be informed that a report is being made.
- Provide information about organizations that offer support to people with experiences of transactional sex, such as Mikamottagning.
- If you suspect a crime, consider filing a police report. See page 66 for more information about when confidentiality can be lifted in the event of a suspected crime.

16. Have you ever given someone compensation for sexual acts in Sweden or abroad?

This includes by telephone or computer. Examples of compensation: money, alcohol, cigarettes, drugs, housing, food, items, or travel. Sexual acts may take place during physical encounters, but they may also involve sharing naked photos, posing or having sex in front of a webcam.

⚠ Yes

✓ No

⚠ I don't know

Background

In Sweden, it is mostly men who pay or give other compensation for sex. Approximately 9% of men and 0.5% of women have at some point given compensation for sex. Homosexual/bisexual and queer men constitute the group that most often give compensation for sex (15%). A large proportion of those who give compensation for sex do so abroad, around 80% (1).

The motives for paying for sexual acts vary; for some, it is related to loneliness and a longing for intimacy and closeness. For others, it is about a sexual activity that they feel they cannot get otherwise due to shyness or social difficulties. In other case, the motives may be related to acting out dominance and power (30, 31).

Different people think and feel differently after paying for sex. Some say it is not a problem. Others feel guilt and shame, which can lead to other health problems such as sleep disturbances, anxiety and other depressive symptoms. One study of men who had paid for sex found that these men were more likely than other men to have been sexually assaulted or physically abused, had a higher number of sexual partners, were more preoccupied with sex, and were more active on the internet for sexual purposes. They also had higher alcohol and drug consumption (32).

In Sweden, it is illegal to give compensation for sex. This applies to all forms of sex with physical contact as well as sexual posing (pictures or films) of people under the age of 18. It is also illegal to arrange contacts, run a brothel or coerce someone to perform sexual acts for compensation (human trafficking).

When children under the age of 18 are offered compensation, this is called commercial sexual exploitation. The crimes are usually categorized as sexual posing, sexual molestation, or sexual assault/rape.

Follow-up questions

What happened?

Have you ever been offered the opportunity to pay for or provide other compensation for sex?

Have you ever considered paying for sexual acts? (Also applies online)

Has it happened that those you gave compensation for sex were under the age of 18?

Have you told anyone about it?

Did you think about it afterwards?

Why did you give compensation for sex?

How do you feel it has affected you?

Have you previously sought help or treatment for this?

Is this something you would like support with?

Actions

- Inform the patient that in Sweden, it is illegal to pay for sex according to the Sex Purchase Act.
- Information and guidance on testing for sexually transmitted diseases. It is a good idea to use the STI sampling template that you can find at https://www.vgregion.se/halsa-och-vard/vardgivarwebben/amnesomraden/kunskapscentrum-for-sexuell-halsa/material-och-publikationer/sti-provtagningsmall/
- STI counseling. Inform the patient about the risk of transmission during various sexual practices, including anal and oral sex.
- Contraceptive counseling.
- Condom counseling. Recommend a "condom tape measure." There are free versions to print online.
- Conversations about other safer sex practices, such as oral sex and dry sex.
- Provide information about PrevenTell or other agencies that offer support regarding unwanted sexuality.
- Provide information about services that offer support to people who have paid for sexual acts, such as KAST and RFSU.
- Consider referral or collaboration with others, such as social services, primary healthcare centers, sexology clinics, or psychiatric services.
- If you suspect that a child under the age of 18 is being harmed, file a report to Child Protective Services. Reporting concerns to CPS can also be considered in cases of previous victimization. If necessary, you can inform other relevant parties in the clinic that the report has been made. In the case of domestic violence, do not inform guardians or other relatives about the report. If there is uncertainty about reporting, it is always possible to consult the social services first. Guardians should, as far as possible, be informed that a report is being made.

- If you suspect a crime, consider filing a police report. See page 66 for more information about when confidentiality can be lifted in the event of a suspected crime.

17. Have you ever been sexually aroused by something that could be harmful to you or others?

Examples are: arousal in response to children, animals, forced sex, exposing one's genitals, peeping on someone, or other non-consensual situations.

⚠ Yes

✓ No

⚠ I don't know

Background

Human sexuality is complex, and it is common to have sexual fantasies about situations that feel dangerous or forbidden. This may include fantasies about dominance, submission and violence. Most people understand that these are just fantasies. Fantasizing about a sexual act does not necessarily mean a desire or plan to act it out. Some people do act out their fantasies with others in sexual role-playing but have a clear understanding of their own and others' limits, for example, people who practice BDSM. At the same time, there are also people who subject others to sexual crimes where consent and understanding are lacking.

When a person says that they may have felt sexual arousal toward something that could be harmful to themselves or others, it is important to explore this in a non-judgmental way. Many fantasies may be just fantasies and may then need to be normalized, for example, by informing the person that it is common to fantasize about themes concerning taboos.

Some people feel confident about what they are sexually attracted toward, while others are more insecure. Most people have a broad sexual attraction pattern, meaning they experience sexual arousal toward a variety of things and situations. Some are sexually aroused by a limited or very specific things/situations, which is usually referred to as an exclusive sexual arousal pattern. It is not possible to control what a person is sexually aroused by. It is also important to know that a person can experience shame related to their sexual attraction patterns. This can involve thinking that it is forbidden to be aroused by norm-breaking situations (e.g., humiliation or role-playing) or by specific stimuli such as particular materials or structures.

Studies have shown that approximately 25% of patients with obsessive-compulsive disorder (OCD) have intrusive sexual thoughts (33). Intrusive thoughts about having a pedophilic arousal pattern are among the most common. Patients with obsessive-compulsive disorder (OCD) do not usually experience sexual desire and attraction towards children, but rather are preoccupied with their anxiety about possibly feeling such attraction (34). It can sometimes be difficult to determine whether a person has experienced sexual arousal or not, as some people have tried to "confirm" their anxiety by inducing fantasies or watching material that they are worried about getting aroused by. However, having a physical response such as lubrication or an erection is not the same as feeling arousal, so these issues need to be carefully reviewed or referred to a healthcare professional who has the competence and experience to diagnose OCD.

Getting a physical response to stimulation can also happen in situations of abuse, but it is not the same as being turned on by the situation; it is a completely automatic reaction. People who have been subjected to sexual abuse, on the other hand, can experience arousal toward situations that are reminiscent of the abuse afterwards, which can be experienced as confusing for the abuse survivor. In

such situations, the person may need help from healthcare professionals to understand what is related to desire and what may be about potential self-harming behaviors.

In 2022, 1903 people were prosecuted for some kind of sexual crime in Sweden. Of these, 272 were prosecuted for rape and 177 for child rape (19). In addition, 326 people were prosecuted for child pornography crimes (people who consumed and/or distributed recorded abuse of children). The number of people committing crimes related to abuse material involving children has increased in recent years (19), but the true number is suspected to be higher. Experiencing arousal in situations where consent cannot be obtained, that is, that the person has a paraphilic (deviant) arousal pattern, is an underreported phenomenon, probably largely due to the stigma attached to having such arousal patterns (35). Studies have shown that people with paraphilic arousal patterns seek help if they are allowed the opportunity to receive care anonymously (36). Previous research has shown that people with paraphilic arousal patterns and concurrent problems with compulsive sexual behavior are overrepresented among those who commit sexual offenses (22).

People with paraphilic arousal patterns are diverse. Some have an exclusive arousal pattern, meaning that they only experience arousal toward situations that are illegal to act out. In such cases, treatment may need to focus on ensuring that the sexual interest is not expressed in illegal ways. Others have a broader arousal pattern and may experience arousal toward situations that are not illegal, in which case, part of the treatment can be directed at solidifying these legal behaviors. Healthcare probably has a vital role in identifying individuals with paraphilic arousal patterns and in providing treatment with the aim of reducing the risk of the individual committing criminal and harmful acts, thereby indirectly contributing to protecting other people from exposure to sexual violence.

A person's sexual arousal pattern is something the person has and not who they are. As the issue is sensitive, it is of particular importance to provide professional and respectful treatment to people suspected of having paraphilic sexual arousal patterns.

Follow-up questions

Is this a question you have concerns about?

Do you have sexual interests that you find embarrassing or unusual?

What kind of situations?

Is it about sexual fantasies?

Is it about sexual acts?

In what way do you feel it has been harmful to yourself/others?

Have you watched material whose legal status you are unsure about, such as recorded child sexual abuse?

Have you previously sought help or treatment for this?

Have you been in contact with the police and/or the Prison and Probation Service?

Actions

- Explore whether the behavior is harmful to the patient or others. If not, normalize as much as possible. Explain that there is a wide diversity in sexuality and sexual behaviors.
- If patients use or distribute abusive material or have committed abusive acts, inform the patient that these acts are criminal. Also, confirm the positive aspects of the patient's disclosure, which indicates a willingness to change.
- If you suspect that a child under the age of 18 is being harmed, file a report to Child Protective Services. Reporting concerns to CPS can also be considered in cases of previous victimization. If necessary, you can inform other relevant parties in the clinic that the report has been made. In the case of domestic violence, do not inform guardians or other relatives about the report. If there is uncertainty about reporting, it is always possible to consult the social services first. Guardians should, as far as possible, be informed that a report is being made.
- If you suspect a crime, consider filing a police report. See page 66 for more information about when confidentiality can be lifted in the event of a suspected crime.
- Consider referral or collaboration with others, such as social services, primary healthcare centers, sexology clinics, or psychiatric services.
- Provide information about PrevenTell or other agencies that offer support regarding unwanted sexuality.

18. Are you, or is someone close to you, concerned about how much alcohol you drink?

⚠ Yes

✓ No

⚠ I don't know

Background

It is important to routinely ask about alcohol use as consumption can negatively affect health in many ways. Just over 16% of the Swedish population consumes alcohol in a risky manner (37).

Many people may experience increased impulsivity under the influence of alcohol, which can lead to risk-taking in several areas. Alcohol also has an anti-anxiety effect, and it is not uncommon to use alcohol in connection with sexual situations to relax. Sexual function is affected by alcohol use in multiple ways; for example, it is common to experience erection problems and difficulty reaching orgasm under the influence of alcohol (38, 39).

Among people who have been sexually abused, it is more common to have risky alcohol consumption compared to people who have not experienced sexual violence (3). There is also a proven association between alcohol exposure and increased sexual risk-taking, although this has been studied primarily in adolescents and young adults (40).

Follow-up questions

What makes you feel worried about your alcohol consumption?

Who is concerned about your alcohol consumption and why?

How does alcohol affect your everyday life?

How does alcohol affect your relationships?

On what occasions do you drink alcohol, and how much do you drink on each occasion?

For what purpose do you drink alcohol?

Have you or someone else been harmed because you drank too much?

Have you previously sought help or treatment for this?

Is your alcohol consumption something you would like help with?

Are there children in the family, or other children who may have been affected by your alcohol consumption?

Actions

- Ask the patient to complete the AUDIT and then compile a Risk Assessment.
- Provide information about alkoholhjälpen.se or other activities that offer support regarding alcohol consumption.
- Consider referral or collaboration with others, for example, social services, addiction clinics, primary healthcare centers, sexology clinics, or psychiatric services.
- If a patient needs care for their alcohol use but refuses to accept such care, compulsory care may be required in accordance with the Swedish Medical Association Act (1988:870) on the Care of Drug Abusers in Certain Cases. Such a report is made to the social services by a physician.
- If you suspect that a child under the age of 18 is being harmed, file a report to Child Protective Services. Reporting concerns to CPS can also be considered in cases of previous victimization. If necessary, you can inform other relevant parties in the clinic that the report has been made. In the case of domestic violence, do not inform guardians or other relatives about the report. If there is uncertainty about reporting, it is always possible to consult the social services first. Guardians should, as far as possible, be informed that a report is being made.

19. Have you taken drugs during the past year?

Examples are: cannabis, amphetamines, heroin, LSD, GHB/ GHL, anabolic steroids, unprescribed narcotic medications or other drugs.

⚠ Yes

✓ No

⚠ I don't know

Background

As with alcohol consumption, it is important to routinely ask about drug use. Drug use and the development of addictive disorders can have serious short- and long-term consequences. Studies have shown that there are strong links between addiction problems and having experienced traumatic events (41).

In Sweden, approximately 8% have used drugs during the past year (42). There are many reasons why a person uses drugs, but what most people have in common is that those who use drugs experience some kind of intoxication. Just as with alcohol, drugs can have an anti-anxiety effect and impair our impulse control. In sexual situations, such effects can lead to greater risk-taking (43, 44). Such risk-taking can lead to one's own and others' sexual boundaries being exceeded. Drugs also have an impact on sexual function; some drugs make sexual practice more difficult, while other drugs are perceived to enhance sexual desire and function.

Chemsex is a term for the use of certain substances in connection with sex. The purpose of chemsex is usually to facilitate, enhance or prolong pleasure. Some examples of substances that can be used for this purpose are methamphetamine, GHB/GHL, cocaine and ketamine. Chemsex is associated with the MSM population (44, 45), but the phenomenon of using drugs to enhance sexual experiences also occurs in other populations. As a healthcare professional, it is good to be aware that most chemsex users do not experience an addiction problem, as many have a functioning everyday life. Furthermore, it is important for healthcare professionals to be aware of the associated risks of spreading infection. Sharing syringes when using intravenous drugs involves an increased risk of hepatitis and HIV infections. As for chemsex, this is also associated with an increased risk of STIs, including HIV transmission, as it is often practiced in contexts where people do not use condoms and in connection with group sex or sex with several different partners at the same time (44, 45).

Follow-up questions

How often do you use drugs?

What drugs do you use?

For what purpose do you use drugs?

How do you pay for your drug use?

Have you used drugs in connection with sex?

How does drug use affect your everyday life and relationships with others?

What are the consequences of your drug use?

Have you previously sought help or treatment for this?

Is this something you would like help with?

Are there children in the family, or other children who may have been affected by your use?

Actions

- Ask the patient to fill out the DUDIT and then make a risk assessment.
- Provide information about droghjälpen.se or other organizations that offer support regarding drug use.
- Consider referral or collaboration with others, for example, social services, addiction clinics, chemsex clinics, primary healthcare centers, sexology clinics, or psychiatric services.
- If a patient needs care for their drug use but refuses to accept such care, compulsory care may be required in accordance with the Swedish Medical Association Act (1988:870) on the Care of Drug Abusers in Certain Cases. Such a report is made to the social services by a physician.
- If you suspect that a child under the age of 18 is being harmed, file a report to Child Protective Services. Reporting concerns to CPS can also be considered in cases of previous victimization. If necessary, you can inform other relevant parties in the clinic that the report has been made. In the case of domestic violence, do not inform guardians or other relatives about the report. If there is uncertainty about reporting, it is always possible to consult the social services first. Guardians should, as far as possible, be informed that a report is being made.
- Inform people who inject drugs about the possibility of receiving a hepatitis B vaccination (free of charge).
- If the patient uses injections, inform them about syringe exchange services.

20. Has anyone ever restricted or controlled you?

This may involve your finances, choice of partner, friends, clothing, employment/daily activities, or other violations of your right to privacy. It may also involve someone refusing to help you or preventing you from getting help with your basic needs.

⚠ Yes

✓ No

⚠ I don't know

Background

Trying to control or limit someone else is a demonstration of power and dominance and may be a form of psychological violence (46). In relationships where violence occurs, the violence often starts with controlling behavior from the violent partner. In close relationships, it is not uncommon for control to also include elements of jealousy. Control is often exercised in the form of questioning or interrogation, where the person being controlled must account for everything they have done, who they have met, and where they have been. Control is not always easy to detect, as it can initially be confused with a partner caring deeply (47).

Sometimes control occurs in a collectivistic context with support from family, relatives, congregations or similar groups. It is the collective perspective that characterizes control within honor-related violence and oppression, where it is most often the sexuality of family members that is controlled so that the family's honor is not tarnished (48). Control can be expressed through restrictions on living space and threats of exclusion. In more extreme cases, honor-based thinking can involve threats of violence, actual violence, or, in the worst case, murder. Other expressions of honor-related violence and oppression can be female genital mutilation, child marriage and coerced marriage (49). Honor-related violence particularly affects young people, LGBTQI people and people with intellectual disabilities (48). Around 5% of young people aged 16-25 live in a context where they are not completely free to choose who they will marry (50).

For people who are dependent on care or support, such as people with disabilities or patients in elderly care, violence can be committed in the form of neglect. Neglect can, for example, mean that the victim does not receive necessary help with food, medicine or hygiene. The victim may also be denied help with basic activities, for example, getting out of bed, or be given too little, too much or the wrong medicine (46).

Follow-up questions

When did it last occur?

What happened?

Who were the people who controlled or limited you?

Has it happened several times? Is there a risk that it will happen again?

How did you react in the situation and/or afterwards?

Do you feel afraid of anyone around you?

Have you told anyone about it?

How has it affected you?

Is this something you would like help with?

Are there children in the family, or other children who may have been affected by the violence?

Actions

- Inform the patient that violence is harmful and that it may be a crime.
- Provide information about the possibility of healthcare, support from social services and contact with voluntary organizations in the local area or online. For example, hedersförtryck.se, Terrafem, Stödlinjen för män, Stödlinjen för transpersoner och Kvinnofridslinjen.
- If the patient consents, help them contact social services.
- If genital mutilation is suspected, refer the patient to a vulvar clinic or gynecological clinic.
- Consider referral or collaboration with others, such as social services, primary healthcare centers, or psychiatric services.
- If you suspect that a child under the age of 18 is being harmed, file a report to Child Protective Services. Reporting concerns to CPS can also be considered in cases of previous victimization. If necessary, you can inform other relevant parties in the clinic that the report has been made. In the case of domestic violence, do not inform guardians or other relatives about the report. If there is uncertainty about reporting, it is always possible to consult the social services first. Guardians should, as far as possible, be informed that a report is being made.
- If you suspect a crime, consider filing a police report. See page 66 for more information about when confidentiality can be lifted in the event of a suspected crime.

21. Have you ever restricted or controlled someone else?

This may involve someone's finances, choice of partner, friends, clothing, employment/daily activities, or other violations of his or her right to privacy. It may also involve refusing to help or preventing someone from getting help with their basic needs.

Yes

No

I don't know

Background

Control involves the restriction of another person's freedom. In violent relationships, it most commonly manifests as regulating where someone goes, whom they see, or what they do. It may also extend to controlling choices of clothing or personal appearance. (47).

Control in honor-related violence and oppression is most often related to controlling a family member's sexuality and choice of partner. Violence in an honor context can be perpetrated by both women and men. It is most often perpetrated by close relatives, but also by other people connected to the oppressed (46). Boys and men can be both the victims and the perpetrators in the same context. They often perpetrate the violence but may also themselves be controlled by other family members to ensure that the violence and oppression continues according to the desired norm and culture (51).

Only a small proportion of those who perpetrate violence against loved ones seek support, help or care. Just as in the case of exposure to violence, questions about the perpetration of violence may need to be asked at several different healthcare visits. Often, the person who has perpetrated violence does not want to acknowledge this and thus denies subjecting others to control. However, if the perpetrator is in a situation where they feel motivated to change, one question can make a big difference (46).

According to the Health and Medical Services Act (2017:30), HSL, the health and medical services do not have any special responsibility for treating perpetrators of violence to end the violent behavior. However, the health and medical services are recognized as a vital entity in reaching the target group. According to the National Board of Health and Welfare, it may be important for the health and medical services to be aware of the risk that patients may engage in violence in close relationships. For example, if there are children involved, a report to Child Protective Services may be necessary.

If a patient needs treatment to end violent behaviors, all healthcare professionals have a responsibility to refer the person to social services and other qualified healthcare providers (46).

When talking to people who have committed violence, it is important to clearly state and define what the person has done and not to minimize the seriousness of the actions or their responsibility for them. But staff should also encourage the positive aspects of the person talking about their experiences and wanting to change their destructive behavior. It is important to convey a sense of hope that support is available. Without an intervention, the violence is likely to be repeated, and it can escalate and become more serious over time.

Follow-up questions

When did it last occur?

What happened?

Has it happened several times? Do you think it will happen again?

Who are you limiting or controlling?

What made you limit or control another person?

Have you told anyone else about this?

Have you previously sought help or treatment for this?

Is this something you would like support with?

Are there children in the family or other children who may have been affected by the violence?

Actions

- Inform the patient that these actions may be criminal. Also confirm the positive aspects of the patient telling their story, which indicates a willingness to change.
- Provide information about the possibility of healthcare, support from social services and contact with voluntary organizations in the local area or online, for example, the anonymous telephone advice service Välj att sluta, and the website **hedersförtryck.se**
- Consider referral or collaboration with others, such as social services, primary healthcare centers, sexology clinics, or psychiatric services.
- If you suspect that a child under the age of 18 is being harmed, file a report to Child Protective Services. Reporting concerns to CPS can also be considered in cases of previous victimization. If necessary, you can inform other relevant parties in the clinic that the report has been made. In the case of domestic violence, do not inform guardians or other relatives about the report. If there is uncertainty about reporting, it is always possible to consult the social services first. Guardians should, as far as possible, be informed that a report is being made.
- If you suspect a crime, consider filing a police report. See page 66 for more information about when confidentiality can be lifted in the event of a suspected crime.

22. Have you ever been subjected to psychological violence or a threat of violence?

This includes by telephone or computer. Examples of psychological violence: being violated, humiliated, threatened, harassed or bullied, or having property destroyed.

Yes

No

I don't know

Background

Psychological violence can affect anyone, regardless of gender, age or background. Individuals can be subjected to such violence by close relatives and friends as well as by colleagues and strangers.

Women are generally more often exposed to psychological violence than men. In the ***Violence and Health*** population survey**,** 20% of women and 8% of men reported having been subjected to repeated and systematic psychological violence by a current or former partner (3). In the SRHR2017 population survey, 12% of women and 7% of men reported that they had been threatened by a partner. Those most exposed to threats from partners were homosexual women (23%) and bisexual women (22%). Exposure decreased with increasing age (1).

Regarding online harassment, certain groups are particularly exposed. In the 16–35 age group, just over 70% report having been subjected to harassment. People with a foreign background are more exposed than others; approximately 60% report having been subjected to harassment. Other vulnerable groups include LGBTQI individuals and people with various forms of disability (52).

Psychological violence can be criminal acts such as threats, harassment and unlawful coercion. However, it also includes acts that are not defined as crimes under current legislation but which, in context, can create a pattern of victimization, such as derogatory comments, financial exploitation, neglect and isolation from friends and family (46). In cases of intimate partner violence, it is common for the victim to convince themselves that the motives given by the perpetrator for their abuse are justified, thus normalizing the actions (53).

According to the Swedish Work Environment Authority, 10% of all employees have been subjected to work-related bullying or offensive discrimination (54).

Research shows that psychological, physical and sexual violence have consequences for health, both in the short and long term. Psychological violence can have consequences as serious as physical violence. Many victims of violence find that psychological abuse, such as humiliation, attempts at isolation and psychological breakdown, is the most difficult to deal with emotionally (53).

It is important to be aware that people who have been exposed to one type of violence are at increased risk of being exposed to other forms of violence.

Follow-up questions

What happened?

When did it happen?

Who subjected you to it?

Has it happened several times? Is there a risk that it will happen again?

How did you react in the situation and/or afterwards?

Have you told anyone about it?

How has it affected you?

Have you previously sought help or treatment for this?

Is this something you would like support with?

Are there children in the family or other children who may have been affected by the violence?

Actions

- Inform the patient that violence is harmful and that it may be a crime.
- Provide information about the possibility of healthcare, support from social services and contact with voluntary organizations in the local area or online. For example, Terrafem, Stödlinjen för män, Stödlinjen för transpersoner and Kvinnofridslinjen.
- If the patient consents, help them contact social services.
- Consider referral or collaboration with others, such as social services, primary healthcare centers, sexology clinics, or psychiatric services.
- If you suspect that a child under the age of 18 is being harmed, file a report to Child Protective Services. Reporting concerns to CPS can also be considered in cases of previous victimization. If necessary, you can inform other relevant parties in the clinic that the report has been made. In the case of domestic violence, do not inform guardians or other relatives about the report. If there is uncertainty about reporting, it is always possible to consult the social services first. Guardians should, as far as possible, be informed that a report is being made.
- If you suspect a crime, consider filing a police report. See page 66 for more information about when confidentiality can be lifted in the event of a suspected crime.

23. Have you ever subjected someone to psychological violence or a threat of violence?

This includes by telephone or computer. Examples of psychological violence: violating, humiliating, threatening, harassing or bullying someone, or destroying their property.

⚠ Yes

✓ No

⚠ I don't know

Background

Knowledge of how common it is to have subjected someone else to violence is limited because questions on this subject are often not included in population surveys. Therefore, the statistics that are available only cover those suspected and prosecuted for crimes. Regarding psychological violence, there is also a great deal of ignorance about which acts are criminal, and the majority are never reported. Examples of crimes that may be relevant are unlawful threats, harassment, unlawful coercion, insults and defamation. They may also include unlawful invasion of privacy, offensive photography, incitement against a group of people, hate crimes or sexual harassment.

Acts of violence can often be diminished by both the victim and the perpetrator. Of those who were victims of crime in a close relationship in 2012, only 3.9% reported the incident or any of the incidents to the police. By far the most common reason for not reporting the incident was that the person considered the incident to be a minor matter (55).

If a patient needs interventions to end the violent behaviors, all healthcare professionals have a responsibility to refer the person to social services or other qualified healthcare providers (46).

When talking to people who have committed violence, it is important to clearly state and define what the person has done and not to minimize the seriousness of the actions or their responsibility for them. But staff should also encourage the positive aspects of the person talking about their experiences and wanting to change their destructive behavior. It is important to convey a sense of hope that support is available. Without intervention, violence is likely to be repeated, and it can escalate and become more serious over time.

Follow-up questions

When did it happen?

What happened?

Who have you subjected to psychological violence?

Has it happened several times? Do you think there is a risk of it happening again?

Have you told anyone else about it?

Have you previously sought help or treatment for this?

Is this something you would like support with?

Are there children in the family or other children who may have been affected by the violence?

Actions

- Explain that violence is a crime. At the same time, confirm the positive aspects of the patient sharing their experiences, which indicate a willingness to change.
- Provide information about the possibility of healthcare, support from social services, and contact with voluntary organizations in the local area or online. For example, the anonymous telephone line Välj att sluta.
- Consider referral or collaboration with others, such as social services, primary healthcare centers, sexology clinics, or psychiatric services.
- If you suspect that a child under the age of 18 is being harmed, file a report to Child Protective Services. Reporting concerns to CPS can also be considered in cases of previous victimization. If necessary, you can inform other relevant parties in the clinic that the report has been made. In the case of domestic violence, do not inform guardians or other relatives about the report. If there is uncertainty about reporting, it is always possible to consult the social services first. Guardians should, as far as possible, be informed that a report is being made.
- If you suspect a crime, consider filing a police report. See page 66 for more information about when confidentiality can be lifted in the event of a suspected crime.

24. Have you ever been subjected to physical violence?

Examples of physical violence: being hit, kicked, shoved, choked, or harmed in some other way.

⚠ Yes

✓ No

⚠ I don't know

Background

In the Swedish National Security Survey, 3.5% of men and 2.1% of women in the age group 16–84 reported being subjected to physical abuse. Regardless of gender, the prevalence is highest in the younger age group and then decreases with age (56).
The majority (80%) of reported assaults against women are committed by someone known to the victim, while men are more often victimized by an unknown perpetrator (58%) (57). Crime statistics only take legal gender into account; therefore, there are no statistics on Swedish transgender persons' exposure to violence.

Intimate partner violence can affect everyone in society, regardless of gender, age or background. Despite this fact, there are strong, incorrect stereotypes about who practices and is subjected to violence in intimate relationships. This probably leads to questions about experiences of violence being exposed not being asked in connection with healthcare visits.

The risk of being exposed to violence in intimate relationships is higher in certain groups, and the violence that women and LGBTQ individuals are subjected to is often more severe and more systematic compared to the violence that men are subjected to. In intimate partner violence, it is common for the violence to escalate gradually, for example, from controlling behaviors and verbal abuse to physical violence. This can lead to expressions of violence being normalized within the relationship and to the victim becoming psychologically broken. This process, together with feelings of shame, guilt and fear, often makes it difficult for the victim of violence to seek care or report it to the police.

Several studies have shown that LGBTQ individuals are a group particularly exposed to violence. This applies to both violence in intimate relationships and the vulnerability that comes with being part of a minority group (3, 58).

Regardless of whether or not the patient reports that violence has occurred, all those who show symptoms or signs that raise suspicion of exposure to violence should be provided with information about the possibilities for care, support and help (59).

Follow-up questions

What happened?

When did it happen?

Who has subjected you to physical violence?

Has it happened several times? Is there a risk that it will happen again?

How did you react? you in the situation? How did you react afterwards?

Have you told anyone about it?

How has it affected you? To what extent do the experiences affect how you feel today?

Is this something you would like support with?

Are there children in the family or other children who may have been affected by the violence?

Actions

- Inform the patient that violence is harmful and that it may be a crime.
- Provide information about the possibility of healthcare, support from social services and contact with voluntary organizations in the local area or online. For example, Terrafem, Stödlinjen för män, Stödlinjen för transpersoner and Kvinnofridslinjen.
- If the patient consents, help them contact social services.
- Consider referral or collaboration with others, such as social services, primary healthcare centers, sexology clinics, or psychiatric services.
- If you suspect that a child under the age of 18 is being harmed, file a report to Child Protective Services. Reporting concerns to CPS can also be considered in cases of previous victimization. If necessary, you can inform other relevant parties in the clinic that the report has been made. In the case of domestic violence, do not inform guardians or other relatives about the report. If there is uncertainty about reporting, it is always possible to consult the social services first. Guardians should, as far as possible, be informed that a report is being made.
- If you suspect a crime, consider filing a police report. See page 66 for more information about when confidentiality can be lifted in the event of a suspected crime.

25. Have you ever subjected someone to physical violence?

Examples of physical violence: hitting, kicking, shoving, choking, or otherwise harming someone.

⚠ Yes

✓ No

⚠ I don't know

Background

As with psychological violence, there is a lack of statistics on people who have committed physical violence, as questions on this issue are often not included in population surveys. Among those who are reported for assault, the majority are men (78%) (18). Norms around, for example, gender, ethnicity and sexuality influence who we see as a potential perpetrator of violence. This means that non-stereotypical perpetrators of violence risk being overlooked to a greater extent.

The national anonymous telephone advice service for people who perpetrate violence, "Choose to quit", has seen an increase in calls from people who seek help for their behavior since the service was launched in 2021. Most people who received a referral to some kind of service that targets perpetrators of violence had previously called the telephone advice service for a consultation. In 2022, 279 calls were received, of which 81% came from men and 19% came from women. In 68% of the calls, other problems that occurred in parallel with the violence were also described, such as general difficulties with relationships, mental health problems and addiction problems (60).

People who commit violence are usually aware that what they are doing is harmful and criminal. Many are ashamed of their behavior and do not want to be identified as perpetrators of violence. Therefore, it is common to try to minimize and justify the behavior, both to themselves and to others. Few seek help of their own accord, but those who do, often use descriptions that can make it unclear that the problem concerns violence. For example, they may describe themselves as being angry, aggressive or having outbursts of anger (61).

When talking to people who have committed violence, it is important to clearly state and define what the person has done and not to minimize the seriousness or responsibility for the actions. But staff should also encourage the positive aspects of the person talking about their experiences and wanting to change their destructive behavior. It is important to convey a sense of hope that support is available. Without intervention, violence is likely to be repeated, and it can escalate and become more serious over time.

If a patient may need interventions to end the violence, all healthcare professionals have a responsibility to refer the person to social services or other qualified healthcare providers (46).

Follow-up questions

When did it happen?

What happened?

Who have you subjected to physical violence?

Has it happened several times? Do you think there is a risk of it happening again?

Have you told anyone about it?

Have you previously sought help or treatment for this?

Is this something you would like support with?

Are there children in the family or other children who may have been affected by the violence?

Actions

- Explain that violence is a crime. At the same time, confirm the positive aspects of the patient sharing their experiences, which indicate a willingness to change.
- Provide information about the possibility of healthcare, support from social services, and contact with voluntary organizations in the local area or online. For example, the anonymous telephone line Välj att sluta.
- Consider referral or collaboration with others, such as social services, primary healthcare centers, sexology clinics, or psychiatric services.
- If you suspect that a child under the age of 18 is being harmed, file a report to Child Protective Services. Reporting concerns to CPS can also be considered in cases of previous victimization. If necessary, you can inform other relevant parties in the clinic that the report has been made. In the case of domestic violence, do not inform guardians or other relatives about the report. If there is uncertainty about reporting, it is always possible to consult the social services first. Guardians should, as far as possible, be informed that a report is being made.
- If you suspect a crime, consider filing a police report. See page 66 for more information about when confidentiality can be lifted in the event of a suspected crime.

26. When you were growing up, did you ever see anyone in your family being subjected to psychological, physical, or sexual violence?

⚠ Yes

✓ No

⚠ I don't know

Background

Experiencing violence in the family as a child is a serious form of psychological abuse. Experiencing or witnessing violence while growing up can have just as serious consequences as if the child is directly exposed to physical, psychological or sexual violence. The risk of developing mental illness increases the more extensive and difficult the experiences the person has been exposed to, and several studies show that exposure to violence in childhood has negative consequences in the form of mental and physical health problems well into adulthood (3, 62).

It is a criminal offense to allow children to witness certain criminal acts in an intimate relationship, such as violence and sexual offenses. Violations against children's integrity are punishable by up to two years in prison, while serious violations can result in a prison sentence of up to four years (46).

Follow-up questions

What happened?

When did it happen?

How long did it go on?

Have you told anyone about it?

How has it affected you?

Is this something you would like support with?

Actions

- Tell them that violence is a crime.
- Provide information about the possibility of healthcare and medical care as well as contact with voluntary organizations in the local area or online.
- Consider referral or collaboration with others, such as social services, primary healthcare centers, sexology clinics, or psychiatric services.
- If you suspect that a child under the age of 18 is being harmed, file a report to Child Protective Services. Reporting concerns to CPS can also be considered in cases of previous victimization. If necessary, you can inform other relevant parties in the clinic that the report has been made. In the case of domestic violence, do not inform guardians or other relatives about the report. If there is uncertainty about reporting, it is always possible to consult the social services first. Guardians should, as far as possible, be informed that a report is being made.

Judicial concerns

Confidentiality

Confidentiality is an important principle in healthcare. If information arises that raises concerns, it is important, in collaboration with the patient, to explore what has happened. It is recommended that the clinic provide information about confidentiality and in which cases it may need to be lifted. The information can be made available, for example, through posters in the waiting room or through verbal information in connection with the healthcare visit.

Reporting in accordance with Chapter 14, Section 1 of the Social Services Act

All staff are obliged to immediately report to Child Protective Services if, in the course of their work, they become aware or suspect that a child (0–18 years) is being harmed. The obligation to report therefore arises when a suspicion arises that something is happening that may require social services to intervene to protect a child. An assessment of whether the social services need to intervene cannot be made by any authority other than the social services themselves.

Anyone considering making a report can consult the local social services office before making the report. This can be done without revealing the child's identity. However, a formal report requires that the healthcare staff have information that allows the child to be identified by the social services. A consultation can never replace a report if there is reason to make one. If there is reason to make a report of concern, a basic principle is that the patient is informed that a report is being made. In urgent cases on weekdays during the day, the social services in the respective municipality/district are contacted. On weekdays after office hours and on weekends, the social services in the respective municipality/district are contacted.

Suspicion of crime

Regarding suspected crimes, the health and medical services have the option, but not the obligation, to file a police report. If the suspected act carries a penalty of at least one year, confidentiality can be lifted without the patient's consent. In the case of an attempted/planned crime, the penalty must be at least two years. To prevent and deter crimes in close relationships, a new confidentiality-lifting provision has been introduced in the Public Access and Secrecy Act, OSL. The act allows staff in the social services and health and medical services, under certain conditions, to provide information to the Police Authority with the aim of preventing serious crimes.

Of course, lifting confidentiality and filing a police report without the patient's consent is not a decision to be taken lightly, and consent should be sought. It is important to always consult with colleagues and the closest manager before making a report. If you are in any doubt, you can call anonymously and consult with the police or prosecutor.

References

1. Sexual and reproductive health and rights (SRHR) in Sweden 2017 - Results from the population survey SRHR2017. Swedish Public Health Agency, 2019.

2. Ryan KL, Arbuckle-Bernstein V, Smith G, Phillips J. Let's Talk About Sex: A Survey of Patients' Preferences When Addressing Sexual Health Concerns in a Family Medicine Residency Program Office. Primer. 2018 Oct 11;2:23. doi: 10.22454/PRiMER.2018.728252. PMID: 32818195; PMCID: PMC7426112.

3. Violence and health. A population survey on women's and men's exposure to violence and the connection to health. National Center for Women's Peace, 2014.

4. National Action Plan for Sexual and Reproductive Health and Rights (SRHR) in Sweden. Implementation of the National SRHR Strategy 2023–2033. Swedish Public Health Agency, 2023.

5. Hammarström S. Identification of young people at risk of sexual ill health: implementing a new tool in youth clinics. Dissertation. Linköping: Linköping University Electronic Press; 2021. Available at: https://urn.kb.se/resolve?urn=urn:nbn:se:liu:diva-178042

6. Starrs AM, Ezeh AC, Barker G, Basu A, Bertrand JT, Blum R, et al. Accelerate progress—sexual and reproductive health and rights for all: report of the Guttmacher–Lancet Commission. The Lancet. 2018;391(10140):2642-92.

7. Advertisement JS. The PLISSIT Model: a proposed conceptual scheme for the behavioral treatment of sexual problems. J Sex Educ Ther 1976;1-15

8. Health determinants for transgender people - a report on the health situation among transgender people in Sweden. Stockholm: Public Health Agency of Sweden, 2011.

9. Blondeel K, Say L, Chou D, Toskin I, Khosla R, Scolaro E, Temmerman M. Evidence and knowledge gaps on the disease burden in sexual and gender minorities: a review of systematic reviews. Int J Equity Health. 2016; 22:15:16.

10. RFSU Condom Quiz 2022. National Association for Sexual Education, 2023.

11. Persson, PA. Safer, Safer, Safest - conversations about safer sex and risk-reducing actions. Gothenburg: Västra Götalandsregionen, 2023.

12. A spectrum of sexual consent - An interview study on consent among sexually active adults. Public Health Agency of Sweden, 2020.

13. Pre-exposure prophylaxis to reduce the risk of HIV infection - A knowledge overview. Swedish Public Health Agency, 2017.

14. Edgardh K, Grünewald M, Kühlmann-Berenzon S, Qvarnström I, Rotzén Östlund M. The perception that “chlamydia is a risk for chlamydia” has been confirmed. Risk for “reinfection” mapped in a prospective cohort study. Läkartidningen 2010;107:3202-7.

15. Duncan B, Hart G, Scoular A, Bigrigg A. Qualitative analysis of psychosocial impact of diagnosis of Chlamydia trachomatis: implications for screening. BMJ 2001; 322:195-9.

16. Kilander H. Contraceptive counseling in abortion care (doctoral thesis). Linköping: Linköping University, 2018.

17. Statistics on abortions 2022, National Board of Health and Welfare, 2023.

18. Suspects 2022 – Final statistics. Stockholm: Swedish Council for Crime Prevention, 2023.

19. Persons prosecuted for crimes 2022 - Final statistics. Stockholm: Swedish Council for Crime Prevention, 2023.

20. Cortoni, F, Babchishin, KM, Rat, C. The proportion of sexual offenders who are female is higher than thought: a meta-analysis. Criminal Justice and Behaviour. 2017; 44(2):145-62.

21. Di Gioia, R, Beslay, L, Cassar, A, Pawula, A. Classification criteria for child sexual abuse and exploitation prevention programmes. Luxembourg: Joint Research Centre; 2022.

22. Hanson, KR, Harris AJR, Scott TL, Helmus, L. Assessing the risk of sexual offenders on community supervision: The Dynamic Supervision Project. Public Safety Canada; 2007.

23. Sexual communication, consent and health – A survey study on how people communicate in sexual situations and what consequences this can have. Public Health Agency of Sweden, 2019.

24. Fredlund C. Adolescents selling sex and sex as self-injury (doctoral thesis). Linköping University, 2018.

25. Kuzma, JM, Black DW. Epidemiology, prevalence, and natural history of compulsive sexual behavior. Psychiatric Clinics of North America. 2008;31(4): 603-11.

26. Sexuality and health among young people in Sweden. UngKAB15 – a study on knowledge, attitudes and behavior among young people aged 16–29. Swedish Public Health Agency, 2017.

27. Ernst, F, Romanczuk-Seiferth, N, Köhler, S, Amelung, T, Betzler, F. Students in the sex industry: Motivations, feelings, risks, and judgments. Frontiers in Psychology. 2021;12: 1-10.

28. Shepp, V, O'Callaghan, E, Kirkner, A, Lorenz, K, Ullman, S. Sexual assault survivors who exchange sex: Identity, stigma, and informal responses from support providers. Affilia. 2020; 35(1):105-28.

29. Deering, KN, Amin, A, Shoveller, J, Nesbitt, A, Garcia-Moreno, C, Duff, et al. A systematic review of the correlates of violence against sex workers. Am J of Public Health. 2014;104(5): 42-54.

30. Grönvall, Y, Holmström, C, Plantin, L. Doing trust work – the purchase of sex in a Swedish context. Sexualities. 2021; 24(4): 654-72.

31. Grönvall, Y, Holmström, C, Plantin, L. The construction of intimacy in long-term commercial relationships in Sweden. Culture, Health and Sexuality. 2022; 24(4): 451-65.

32. Priebe G, Svedin CG. Selling and buying sex in Sweden 2011. Prevalence, health and attitudes. Interim report 1 from Prostitution in Sweden – Mapping and evaluation of prostitution groups' efforts and experiences and attitudes in the population. Linköping: Linköpings Universitet, 2012.

33. Grant, JE, Pinto, A, Gunnip, M, Mancebo, MC, Eisen, JL, Rasmussen, SA. Sexual obsessions and clinical correlates in adults with obsessive-compulsive disorder. Comprehensive Psychiatry. 2006; 47:325-9.

34. Leavell Bruce, S, Ching, THW, Williams, MT. Pedophilia-themed obsessive–compulsive disorder: Assessment, differential diagnosis, and treatment with exposure and response prevention. Arch Sex Needs. 2018; 47:389-402.

35. Jahnke, S. The stigma of pedophilia: clinical and forensic implications. European Psychologist. 2018; 23(2): 144-53.

36. Lätth, J, Landgren, V, McMahan, A, Sparre, C, Eriksson, J, Malki, K et al. Effects of internet-delivered cognitive behavioral therapy on use of child sexual abuse material: A randomized placebo-controlled trial on the Darknet. Internet Interventions. 2022; 30.

37. Public Health Agency of Sweden. Alcohol consumption in different groups of the population [Internet], retrieved from https://www.folkhalsomyndigheten.se/livsvillkor-levnadsvanor/andts/utveckling-inom-andts-anvandning-och-ohalsa/anvandning/alkoholkonsumtion/alkoholkonsumtion-i-befolkningen/ Updated 2023-06-30, retrieved 2023-08-08.

38. Li, S, Song, JM, Zhang, K, Zhang, CL; A Meta-Analysis of Erectile Dysfunction and Alcohol Consumption. *Urol Int.* 29 October 2021;105 (11-12):969–985. doi: https://doi.org/10.1159/000508171

39. Salari N, Hasheminezhad R, Almasi A, Hemmati M, Shohaimi S, Akbari H, Mohammadi M. The risk of sexual dysfunction associated with alcohol consumption in women: a systematic review and meta-analysis. BMC Women's Health. 2023 May 2;23(1):213. doi: https://doi.org/10.1186%2Fs12905-023-02400-5.

40. Strandberg, A, Kvillemo, P. Alcohol, drugs and sexual health. Stockholm: STAD; 2017. Report no. 61.

41. Simmons, S, Suárez, L. (2016). Substance Abuse and Trauma. *Child and adolescent psychiatric clinics of North America*, 2016;*25*(4),723–734. https://doi.org/10.1016/j.chc.2016.05.006

42. The Swedish National Association for Alcohol and Drug Education, CAN. Use and addiction problems of alcohol, drugs and tobacco. 2022, CAN report 209.

43. Miltz AR, Rodger AJ, Sewell J, Gilson R, Allan S, Scott C, Sadiq T, Farazmand P, McDonnell J, Speakman A, Sherr L, Phillips AN, Johnson AM, Collins S, Lampe FC; AURAH and ASTRA Study Groups. Recreational drug use and use of drugs associated with

chemsex among HIV-negative and HIV-positive heterosexual men and women attending sexual health and HIV clinics in England. Int J Drug Policy. 2021 May;91:103101. doi: https://doi.org/10.1016%2Fj.drugpo.2020.103101

44. Maxwell, S., Shahmanesh, M., & Gafos, M. (2019). Chemsex behaviors among men who have sex with men: A systematic review of the literature. *The International journal on drug policy*, 2019;*63*:74–89. https://doi.org/10.1016/j.drugpo.2018.11.014

45. Tomkins A, George R, Kliner M. Sexualised drug taking among men who have sex with men: a systematic review. Perspective Public Health. 2019 Jan;139(1):23-33. doi: https://doi.org/10.1177/1757913918778872

46. National Board of Health and Welfare. Violence in close relationships - Handbook for social services, health care and dental care. National Board of Health and Welfare, 2023.

47. Isdal P. The meaning of violence. Stockholm: Gothia Publishing House, 2001.

48. National Board of Health and Welfare. Assessment of honour-related violence and oppression and violence in close relationships - Support for social services and healthcare. National Board of Health and Welfare, 2021.

49. Action plan to combat men's violence against women, honour-related violence and oppression, and violence in same-sex relationships. Government letter 2007/08:39.

50. Married against her will. Swedish Youth Agency, 2009.

51. Schlytter, A, Rexvid, D. Men's honor. Being both victim and perpetrator. Valmiera: Student Literature; 2017.

52. Svensson, M, Björkenfeldt O, Åström, F, Dahlstrand, K. Cybercrime and democratic participation – a knowledge overview. Umeå: Crime Victims' Agency, 2021.

53. National Center for Women's Peace. Expressions and Mechanisms of Violence [Internet]. Uppsala: National Center for Women's Peace; 2023 [Updated 2023-05-23, cited 2023-08-04] Retrieved from: https://www.nck.uu.se/kunskapsbanken/amnesguider/vald-i-nara-relationer/valdets-uttryck-och-mekanismer/

54. Järvholm, B. Work-related deaths in Sweden - An update of the Swedish Work Environment Authority's knowledge compilations 2019:3/4. Umeå: Umeå University; 2023, ISSN no. 2003-328.

55. Swedish Council for Crime Prevention, Brå (2014). Crime in close relationships. A national survey. Report 2014:8. Stockholm: Swedish Council for Crime Prevention.

56. National Security Survey 2022. Stockholm: Swedish Council for Crime Prevention, report 2022:09.

57. Reported crimes 2022 – final statistics. Stockholm: Swedish Council for Crime Prevention, 2023.

58. Violence against LGBTQ people – A research and knowledge overview, National Center for Women's Peace, 2018.

59. The National Board of Health and Welfare's regulations and general advice on violence in close relationships. HSLF-FS 2022:39.

60. County Administrative Board. Choose to stop – A telephone line for people who practice violence in close relationships. Final report for the financial year 2022. County Administrative Board, Report 2023:4.

61. Boethius, S. (2015). Men, violence and moral work: Reports from men who have sought treatment for violence in close relationships. [Doctoral dissertation (monograph), Department of Sociology]. Lund University.

62. Felitti VJ, Anda RF, Nordenberg D, Williamson DF, Spitz AM, Edwards V, et al. (May 1998). "Relationship of childhood abuse and household dysfunction to many of the leading causes of death in adults. The Adverse Childhood Experiences (ACE) Study". *American Journal of Preventive Medicine*. **14** (4): 245–258. doi:10.1016/S0749-3797(98)00017-8

Glossary

LGBTQIA+ is a collective term for people who are homosexual, bisexual, trans, queer, intersex, asexual and/or have other identities or sexual orientations.

Homosexual – a person who falls in love with and/or is attracted to people of the same sex.

Bisexual – a person who falls in love with and/or is attracted to people regardless of gender.

Transgender – an umbrella term for people whose gender identity and/or gender expression does not match the legal sex assigned at birth.

Queer – a term for people who break norms regarding gender, sexuality and/or how one forms relationships.

Intersex – a person whose body at birth does not anatomically follow prevailing gender norms, for example, regarding sex chromosomes or genitals. Not to be confused with transgender.

Asexual – a person who does not experience sexual attraction or interest in sex.

Cisperson – a person who identifies with the legal gender assigned at birth.

Compulsive sexuality/compulsive sexual behavior disorder – a clinical condition in which a person has severe sexual problem behaviors that lead to distress and/or negative consequences in everyday life.

Gender-affirming care – care that can be provided to people diagnosed with gender dysphoria to reduce dysphoria and confirm the gender the person identifies with. Gender-affirming care can include hormone therapy, surgery, or voice training.

Gender dysphoria – when the experience that one's gender identity does not match the legal sex assigned at birth leads to suffering. Gender dysphoria can also be a diagnosis.

MSM – men who have sex with men.

OCD – obsessive-compulsive disorder is a clinical condition in which a person experiences obsessions/compulsions. The obsessions lead to anxiety that the person may try to relieve by performing compulsive actions.

Paraphilic arousal pattern – deviant arousal patterns, such as experiencing arousal in non-consensual situations.

PrEP – pre-exposure prophylaxis is a medication taken as a preventive measure to reduce the risk of getting HIV.

Sexual response – automatic physical responses that are linked to sexuality but do not necessarily involve sexual desire, such as lubrication or erection.

STIs – sexually transmitted infections such as gonorrhea, HIV and chlamydia.

Sugar dating – a term for dating that takes place between an older person and a younger person, which involves compensation from the older person, for example, for socializing or sex.

Transition – is when a transgender person makes changes to live more in accordance with their gender identity. Transition can involve changing their name and pronouns or undergoing various physical changes with gender-affirming care.
