# Supplementary material for: Identifying sexual health concerns, risk-taking and violence using the newly developed SEXIT Adult tool in a Swedish public outpatient sample
Source: BMC Public Health. 2026 Jul 30;26:2369. doi: 10.1186/s12889-026-28764-5 (PMC13476919; doi:10.1186/s12889-026-28764-5)
Supplement: Supplementary file 3 [file 12889_2026_28764_MOESM3_ESM.pdf]

Table of contents for the SEXIT Adult training course

Full-day course (between 09:00 and 16:00, 1 hour lunch break 12:00-13:00)

- Purpose of the SEXIT Adult research project
- Research method
- Introduction and purpose of the SEXIT Adult method
- Administration of the SEXIT Adult questionnaire
- Introduction to working with sexual health: the PLISSIT-model
- Sexuality and norms
- Workshop activity 1: Challenging one's own norms around sexuality
- Judicial aspects of sexual health and violence: Confidentiality, Social services and suspected criminal acts
- Dealing with difficult patient reactions: suicidal ideation, trauma response and acute distress.
- Introduction to the SEXIT Adult handbook
- Guidance and recommendations on how to deal with the responses on the SEXIT Adult questionnaire
- Introduction to each question in the SEXIT Adult questionnaire: Background and recommendations
- Workshop activity 2: Reflecting on follow-up questions and further management of three different fictive patient cases.
- Workshop activity 3: Practical training with filled out SEXIT Adult questionnaires, reflection on possible follow-up questions, overall assessment and further management.
- Practical research routines for participating clinics.
- Ongoing support in research routines: contact information to head researcher.
- Ongoing support in patient consultations: contact information to specialist psychologist.
